# Bioinspired One Cell Culture Isolates Highly Tumorigenic and Metastatic Cancer Stem Cells Capable of Multilineage Differentiation

**DOI:** 10.1002/advs.202000259

**Published:** 2020-04-28

**Authors:** Hai Wang, Pranay Agarwal, Bin Jiang, Samantha Stewart, Xuanyou Liu, Yutong Liang, Baris Hancioglu, Amy Webb, John P. Fisher, Zhenguo Liu, Xiongbin Lu, Katherine H. R. Tkaczuk, Xiaoming He

**Affiliations:** ^1^ Fischell Department of Bioengineering University of Maryland College Park MD 20742 USA; ^2^ CAS Key Laboratory for Biomedical Effects of Nanomaterials & Nanosafety CAS Center for Excellence in Nanoscience National Center for Nanoscience and Technology Beijing 100190 China; ^3^ University of Chinese Academy of Sciences Beijing 100049 China; ^4^ Department of Biomedical Engineering The Ohio State University Columbus OH 43210 USA; ^5^ Division of Cardiovascular Medicine Center for Precision Medicine University of Missouri School of Medicine Columbia MO 65212 USA; ^6^ Department of Biomedical Informatics The Ohio State University Columbus OH 43210 USA; ^7^ Department of Medical and Molecular Genetics and Melvin and Bren Simon Cancer Center Indiana University School of Medicine Indianapolis IN 46202 USA; ^8^ Marlene and Stewart Greenebaum Comprehensive Cancer Center University of Maryland Baltimore MD 21201 USA; ^9^ Robert E. Fischell, Institute for Biomedical Devices University of Maryland College Park MD 20742 USA

**Keywords:** cancer stem cells, differentiation, drug resistance, metastasis, microfluidics

## Abstract

Cancer stem cells (CSCs) are rare cancer cells that are postulated to be responsible for cancer relapse and metastasis. However, CSCs are difficult to isolate and poorly understood. Here, a bioinspired approach for label‐free isolation and culture of CSCs, by microencapsulating one cancer cell in the nanoliter‐scale hydrogel core of each prehatching embryo‐like core–shell microcapsule, is reported. Only a small percentage of the individually microencapsulated cancer cells can proliferate into a cell colony. Gene and protein expression analyses indicate high stemness of the cells in the colonies. Importantly, the colony cells are capable of cross‐tissue multilineage (e.g., endothelial, cardiac, neural, and osteogenic) differentiation, which is not observed for “CSCs” isolated using other contemporary approaches. Further studies demonstrate the colony cells are highly tumorigenic, metastatic, and drug resistant. These data show the colony cells obtained with the bioinspired one‐cell‐culture approach are truly CSCs. Significantly, multiple pathways are identified to upregulate in the CSCs and enrichment of genes related to the pathways is correlated with significantly decreased survival of breast cancer patients. Collectively, this study may provide a valuable method for isolating and culturing CSCs, to facilitate the understanding of cancer biology and etiology and the development of effective CSC‐targeted cancer therapies.

## Graphical abstract

A bioinspired technology is developed to isolate cancer stem cells (CSCs) by culturing one cell in the nanoliter‐scale hydrogel core of the prehatching embryo‐like core–shell microcapsules. The CSCs are capable of cardiac, neural, osteogenic, and endothelial differentiation, and are highly tumorigenic, metastatic, and drug resistant. The technology may be valuable for understanding cancer biology/etiology and developing CSC‐targeted effective cancer therapies.

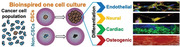

## 1 Introduction

There is mounting evidence that suggests a small subset of cancer cells possesses the exclusive capability of forming tumors, and these cells are often called cancer stem cells (CSCs) or tumor initiating cells.^[^ ^1^ ^]^ CSCs have been posited to be responsible for the failure of the widely used chemo‐ and radiotherapies of cancer due to their drug resistance and high capability of tumorigenesis and metastasis, and cancer treatments could be made more effective by targeting and killing the CSCs.^[^ ^2^ ^]^ Unfortunately, the CSCs are elusive, and their biology is poorly understood up to date. Therefore, establishing a reliable approach to isolate and culture CSCs is invaluable for not only improving our understanding of the CSCs but also developing effective therapeutic strategies for cancer therapy via targeting the CSCs.

“CSCs” have been isolated based on surface markers, such as CD44, CD133, CD24, epithelial cell adhesion molecule (EpCAM), aldehyde dehydrogenase 1 (ALDH1), and adenosine triphosphate (ATP)‐binding cassette B5 (ABCB5).^[^ ^3^ ^]^ This isolation method often causes confusion. This is because two or more surface makers have been used to identify CSCs from the same type of cancer in different studies, but co‐expression of the different surface markers among the selected CSCs is limited.^[^ ^4^ ^]^ For example, either ALDH1^+^ or CD44^+^CD24^−/low^ has been used to select CSCs of breast cancer, but a surprisingly small percentage (0.1–1.2%) of the CSCs expresses both markers simultaneously.^[^ ^5^ ^]^ For pancreatic cancer, either CD44^+^CD24^+^ESA(epithelial‐specific antigen)^+^ or CD133 has been used to select its CSCs, while only 10–40% of the CD44^+^CD24^+^ESA^+^ cells in primary tumors are shown to be positive for CD133 expression.^[^ ^6^ ^]^ Similarly, the EpCAM^+^CD44^+^ colorectal CSCs exhibit little overlap with the CD133^+^ population.^[^ ^7^ ^]^ In essence, it appears that none of these markers are consistently expressed on the solid tumor CSCs and the specific CSC marker(s) for a given type of cancer is still unknown.

Another widely used method for obtaining “CSCs” is to enrich them with suspension culture in defined CSC medium without serum.^[^ ^8^ ^]^ Although hanging drops,^[^ ^9^ ^]^ gyratory rotation and spinner flask,^[^ ^10^ ^]^ and NASA rotary cell culture systems^[^ ^11^ ^]^ have been developed to enrich CSCs via suspension culture, ultralow attachment plates (ULAPs) are most commonly used to enrich CSCs in suspension for various types of cancers.^[^ ^12^ ^]^ The hypothesis is that only CSCs could survive and form cell aggregates/spheroids, while non‐CSCs should die of anoikis during the suspension culture. To investigate CSC isolation by this method, four human cancer cell lines (MDA‐MB‐231, PC‐3, MCF‐7, and OVCAR‐8 cancer cells) were cultured with the ULAP method to evaluate their capability of forming spheroids. As shown in Figure S1a–d (Supporting Information), the MDA‐MB‐231 triple negative human breast cancer cells and PC‐3 human prostate cancer cells can form some loose aggregates, while the MCF‐7 human epidermal growth factor (EGF) receptor 2 (HER2)^+^ human breast cancer cells and OVCAR‐8 human ovarian cancer cells can form tight cell spheroids under the ULAP culture. We do not call the aggregates of MDA‐MB‐231 and PC‐3 cells as spheroids because the aggregates can be easily detached/dissociated into single cells by gentle pipetting for 5 times. Furthermore, we cultured the four types of cancer cells at 1–20,000 cells per well in the ULAP. Surprisingly, we noticed that more than 90% of the cells could survive and grow into aggregates or spheroids under the suspension culture. This suggests that the suspension culture method for CSC isolation based on the anoikis of non‐stem cancer cells is questionable as CSCs are considered to be rare in the cancer cell population. Taken together, the CSCs remain elusive today and it is challenging and confusing to isolate and/or culture them with the contemporary approaches based on surface marker(s) and/or simple suspension culture. Therefore, the development of a marker‐free method for effective isolation and culture of true CSCs is in need.

## 2 Results and Discussion

### 2.1 Bioinspired one cell culture for isolating CSCs

**Figure 1:**
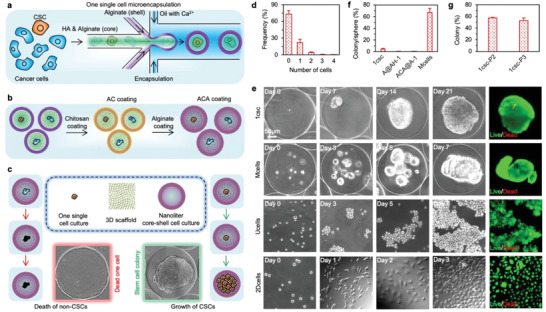
The bioinspired one single cell microencapsulation for isolation and culture of cancer stem cells (CSCs). a) A schematic illustration of the one single cell microencapsulation using a nonplanar microfluidic system. Alginate (A) and hyaluronan (HA or H) are used to form the AH core scaffold while the shell is formed with alginate hydrogel. The one single cell is located in the core of the core–shell microcapsule. b) To keep the HA (H) inside the core–shell microcapsules, the outer surface of the alginate (A) shell is coated with chitosan (C) first and then alginate (A) through electrostatic interactions to form an ACA coating on the surface, resulting in the ACA@AH core–shell microcapsules. c) A schematic illustration of the one single cell microencapsulation for isolating and culturing CSCs in defined CSC medium: only the CSCs could survive and proliferate in the 3D AH core scaffold of the miniaturized core–shell microcapsules, while the non‐stem cancer cell should die of anoikis. d) Histogram showing the distribution of empty microcapsules and microcapsules with 1–4 cells after microfluidic encapsulation. We have not seen microcapsules with more than 4 cells under the condition used in this study. e) Typical micrographs showing the morphology and growth of one (1) single MDA‐MB‐231 triple negative human breast cancer cell in the ACA‐coated (by default) core–shell microcapsule with HA (H) in the core (ACA@AH‐1 or 1csc), multiple MDA‐MB‐231 cells (10–15 cells per microcapsule) in the ACA‐coated core–shell microcapsules with HA in the core (Mcells), MDA‐MB‐231 cells under conventional suspension culture in ultralow attachment plate (Ucells), and MDA‐MB‐231 cells under 2D culture (2Dcells). f) Approximately 4.4% of the MDA‐MB‐231 cells could form colonies (note: a cell colony refers specifically to a cell spheroid grown from one cell in this study) under the 1csc (ACA@AH‐1) culture. Almost no one single MDA‐MB‐231 cell could survive in either the core–shell microcapsules without ACA coating but with HA in the core (A@AH‐1) or the ACA‐coated core–shell microcapsules without HA in the core (ACA@A‐1). However, cell spheroid could be seen in most of the ACA@AH microcapsules encapsulated with multiple (M) MDA‐MB‐231 cells (Mcells) after 7 days of culture. MDA‐MB‐231 cells under the Ucells culture could form loose aggregates in 7 days while the cells under the 2Dcells culture could attach on the 2D substrate and proliferate to ∼80–90% confluency in 3 days. g) Serial passaging assay showing more than 50% of the cells in the 1csc colonies could form new generations of colonies under the 1csc culture. The first‐passage 1csc (1csc‐P1) culture is the same as the 1csc group in (e, f), while 1csc‐P2 and 1csc‐P3 represent the second‐ and third‐passage 1csc culture using single cells dissociated from the 1csc‐P1 and 1csc‐P2 colonies, respectively.

To address the aforementioned challenges and efficiently isolate and culture CSCs, we have been inspired by the nature's approach of culturing stem cells in the prehatching embryos, which starts from one cell (zygote) that proliferates into a cell colony (morula) in a miniaturized (nanoliter) core surrounded by a shell known as the zona pellucida.^[^ ^13^ ^]^ More specifically, we fabricated miniaturized, three‐dimensional (3D), prehatching embryo‐like, core–shell microcapsules to encapsulate one single cancer cell in the nanoliter‐scale hydrogel core of each of the microcapsules for CSC isolation and culture. This mimics the formation of stem cell colony (i.e., morula) from one cell (i.e., zygote) in the prehatching embryo. To achieve this, a microfluidic device was used to fabricate the one cancer cell‐laden core–shell hydrogel microcapsules with a core diameter of 206.5 ± 19.7 µm and shell thickness of 40.5 ± 14.2 µm (**Figure** **1**a and Figure S2, Supporting Information). Since recent studies show that the 3D hydrogel/scaffold may induce anoikis of non‐stem cancer cells,^[^ ^14^ ^]^ we applied an alginate‐based hydrogel scaffold in the core of the microcapsules. Alginate is used because it is highly biocompatible and does not have cell adhesion molecules.^[^ ^15^ ^]^ The latter is good for inducing anoikis of non‐stem cancer cells. To optimize the concentration of alginate, the aforementioned MDA‐MB‐231, MCF‐7, PC‐3, and OVCAR‐8 cancer cells were cultured in alginate hydrogel scaffolds with concentrations ranging over 0.5–3 wt%. As shown in Figure S3a–d (Supporting Information), few cancer cells can proliferate or survive in the 2% and 3% alginate hydrogels. Therefore, 2% alginate was utilized as the core scaffold of the microcapsules to induce anoikis of non‐stem cancer cells. Furthermore, hyaluronan (HA, 0.5 wt%) was embedded in the core as it could play a key role in the CSC niche.^[^ ^16^ ^]^ The shell of the microcapsules was fabricated with pure alginate hydrogel (2%, Figure 1a). A stable core–shell structure was observed in the resultant microcapsules with an overall size of ∼300 µm (Figure S4a, Supporting Information). However, the HA (labeled with fluorescein isothiocyanate or FITC in short) may gradually diffuse out of the microcapsules during 5 days of incubation in medium. To overcome this, we further incubate the microcapsules sequentially with chitosan and alginate (Figure 1b), to form an alginate‐chitosan‐alginate (ACA) coating (Figure S4b, Supporting Information, where the alginate in the ACA coating is labeled with FITC) on the microcapsule surface for reducing the shell permeability. This effectively prevents the leaking of HA from the microcapsule core (Figure S4c, Supporting Information) although the coatings are very thin and do not significantly change the overall size of the microcapsules. By combining the bioinspired one single cell culture and miniaturized 3D hydrogel scaffold selection (Figure 1c), we hypothesized that only the one single CSC in the ACA‐coated microcapsules consisting of a core scaffold of both alginate and HA and an ACA‐coated alginate shell (ACA@AH: A for alginate, H for HA, and C for chitosan) could survive and proliferate during culture.

To test the hypothesis, we quantified the efficiency of obtaining one single cell in the microcapsules after microfluidic encapsulation first. As shown in Figure 1d and Figure S5 (Supporting Information), ∼20% of the ACA@AH microcapsules contained one single cell, whereas ∼4% of the microcapsules contained two or more cells (which were removed from the samples immediately by pipetting). Furthermore, viability of the one single MDA‐MB‐231 cell in the ACA@AH microcapsule is greater than 90% (Figure S6a, Supporting Information) and the cell could attach and proliferate on the regular cell culture plate after releasing them out of the microcapsules by dissolving the alginate hydrogel in the microcapsules with an isotonic solution of sodium citrate and pipetting (Figure S6b, Supporting Information). The one single cell cultured in each of the ACA@AH microcapsule in CSC medium (1csc culture) could form a cell colony (note: we use the term colony only for spheroid grown from one cell) in 21 days (Figure 1e). The cell proliferates slowly during the first and the last 7 days, but fast during days 7–14 (Figure S6c, Supporting Information). Unlike the MDA‐MB‐231 cell aggregates obtained from the conventional ULAP culture (Ucells, Figure 1e) that easily dissociate into single cells after pipetting for 5 times (Figure S1a, Supporting Information), colonies obtained from the 1csc culture are stable even after dissolving the microcapsules with sodium citrate and pipetting for 10 times (Figure S6d, Supporting Information). Importantly, only ∼4.4% of the MDA‐MB‐231 cells under the 1csc culture could survive and proliferate to form a colony (Figure 1f). However, essentially no colonies could be observed either in the absence of the ACA coating (A@AH‐1) to keep HA inside the microcapsules or without HA in the core alginate hydrogel scaffold (ACA@A‐1), confirming that HA plays a pivotal role in the survival and proliferation of CSCs (Figure 1f). However, when encapsulating multiple MDA‐MB‐231 cells in the ACA@AH microcapsules (∼10–15 cells per microcapsules, Mcells in short, Figure 1e), cell spheroids are observed in more than 67% of the microcapsules after only 7 days of culture (Figure 1f). This suggests that the bioinspired use of one single cell is crucial for CSC isolation.

The self‐renewal/stemness of the survived cells is then studied with the serial passaging assay first, for which detached cells obtained by dissociating the first‐passage 1csc (1csc‐P1 or 1csc by default) colonies were encapsulated in the ACA@AH microcapsules (one cell in each microcapsule) for culture to form the second‐passage (1csc‐P2) colonies. Strikingly, more than 50% of the encapsulated one single cell can survive and proliferate to form colonies (Figure 1g and Figure S7, Supporting Information). Similar results are obtained when single cells dissociated from the 1csc‐P2 colonies are encapsulated in the ACA@AH microcapsules (one cell in each microcapsule) for culturing to form the third‐passage (1csc‐P3) colonies (Figure 1g and Figure S7, Supporting Information). These serial passaging data suggest that the cells isolated with the one single cell culture method (i.e., 1csc culture) are likely CSCs. The capability of isolating CSCs with the 1csc culture in the ACA@AH microcapsule is further confirmed using PC‐3, MCF‐7, and OVCAR‐8 cells: ∼7.0%, 5.5%, and 4.2% of the cells under the 1csc culture are able to survive and proliferate into colonies, respectively (Figure S8, Supporting Information).

### 2.2 Gene and protein expression analyses

**Figure 2:**
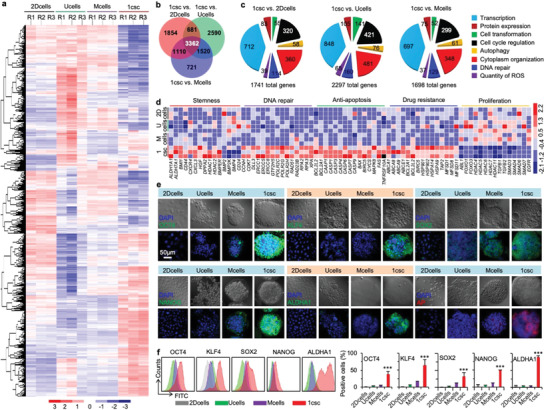
Characterization of stemness with gene and protein expressions. a) Differential gene expression heat map from RNA‐sequencing (RNA‐Seq) of cells obtained from the 2Dcells, Ucells, Mcells, and 1csc cultures, showing the gene expression in the 1csc group is largely different from the other three groups. b) Venn diagram for the differential gene expression heat maps of 2Dcells, Ucells, Mcells, and 1csc groups. c) Gene ontology (GO) enrichment analysis of significantly altered genes in cells obtained from the 1csc versus 2Dcells, Ucells, and Mcells cultures. The whole‐genome data are representative of three independent experiments. d) Heat map of gene expression in cells of the 2Dcells, Ucells, Mcells, and 1csc groups regarding stemness (including four downregulated differentiation marker genes), DNA repair, anti‐apoptosis, drug resistance, and cell proliferation. e) Confocal images of OCT4, KLF4, SOX2, NANOG, ALDHA1, and AP (alkaline phosphatase) protein expression in cells of the 2Dcells, Ucells, Mcells, and 1csc groups. DAPI stains the cell nuclei. f) Representative flow cytometry peaks and quantitative analyses of the expression of OCT4, KLF4, SOX2, NANOG, and ALDHA1 proteins in cells of the 2Dcells, Ucells, Mcells, and 1csc groups. The *p* values for the 1csc group versus the 2Dcells, Ucells, and Mcells groups are <0.0001 for OCT4, <0.0001 for KLF4, 0.0009 for SOX2, <0.0001 for NANOG, and <0.0001 for ALDHA1. Error bars denote mean ± s.d., and statistical significance was assessed by one‐way ANOVA with post hoc Tukey test. ***p < 0.001.

To gain a transcriptome‐wide perspective of alterations in cellular characteristics including stemness in response to the different methods for CSC isolation, we performed RNA sequencing (RNA‐Seq) analyses on the cells in the colonies, spheroids, and aggregates obtained from the 1csc, Mcells, and Ucells culture of MDA‐MB‐231 cells in CSC medium, together with cells under 2D culture in non‐CSC (or regular) medium (2Dcells, Figure 1e). Results obtained from RNA‐Seq were further validated by performing RT‐PCR for six differentially expressed genes (Figure S9a, Supporting Information). First, we confirmed correlation between the four sets of experiments by developing a Pearson correlation matrix (Figure S9b, Supporting Information). All the triplicates are tightly and positively correlated (the diagonal of the heat map, correlation value: 0.97–0.99) and the four culture methods also exhibit high degree of positive correlation (>0.93). This is not surprising as the cells studied in the four groups are all derived from the same cell line. Next, to identify differentially expressed genes, the volcano plots for the four culture methods are assembled by plotting the negative log10 of the *p* value on the y‐axis, where highly significant changes appear high on the plot (Figure S9c, Supporting Information). The expression of ∼37,000 genes in the cells of the 2Dcells, Ucells, Mcells, and 1csc groups was analyzed. The results show that the 1csc group has the highest number of differentially expressed genes when compared with the other three groups (Figure S9b, Supporting Information). A clustergram of the top 10,000 genes that are differentially expressed among the four groups is shown in **Figure** **2**a. In addition, unsupervised hierarchical clustering leads to their organization into distinct clusters. The 1csc group shows significantly greater changes in its transcriptional profiles than the other three groups. Specifically, 7007 (summation of numbers in the red circle), 8153 (summation of numbers in the green circle), and 6713 (summation of numbers in the purple circle) genes were differentially expressed in the 1csc group compared to the 2Dcells, Ucells, and Mcells groups, respectively, as shown by the Venn diagram in Figure 2b and Figure S9c (Supporting Information). A total of 3362 genes were differentially expressed in the 1csc group compared to all the other three groups.

Gene ontology (GO) enrichment analysis was also conducted to determine the significantly altered genes in different biological processes. The top eight enriched GO terms are presented in Figure 2c. It shows that the biological processes related to cellular structure organization (chromatin organization/transcription, cytoplasm organization, and cell transformation), cell proliferation (cell cycle regulation and autophagy), and cellular stress‐related signals (DNA repair and quantity of reactive oxygen species, ROS) are significantly different in the 1csc group compared to all other groups (Figure 2c). The expression of genes that are relevant to stemness (positive and negative) of CSCs, DNA repair, anti‐apoptosis, and drug resistance in the 1csc group compared to all other groups, is shown in Figure 2d. Known functional markers of self‐renewal (*CD44* and *BMI1*) and malignancy (*ALDH1A1, ALDH7A1*) are significantly upregulated in the 1csc group (Figure 2d). Expression of genes (*CXCR4*, *CXCL3*, and *HGF*) involved in secretion of chemokines/cytokines in the tumor microenvironment is significantly upregulated in the 1csc group, as well (Figure 2d). In addition, genes for maintaining pluripotency (e.g., *DPPA2*, *HDAC1*, *HDAC2*, and *BMPER*) are abundantly expressed in the 1csc group. In contrast, genes (*BMP2*, *BMP2K*, *BMP4*, and *CD24*) that promote differentiation and cellular proliferation are downregulated in the 1csc group (Figure 2d). It is worth noting that some key genes (*OCT4*, *SOX2*, *NANOG*, and *KLF4*) important for the maintenance of pluripotency are not upregulated in the 1csc group. However, the expressions of these proteins are high in the 1csc group according to the confocal microscopy (Figure 2e) and flow cytometry analyses with immunofluorescence staining (Figure 2f and Figure S10, Supporting Information). This is probably because the translation of mRNAs into proteins is regulated by many post‐transcriptional processes in cells.^[^ ^17^ ^]^ Indeed, further analyses indicate that many repressors and activators associated with these four stemness genes are downregulated and upregulated, respectively, in the 1csc group (Figure S11, Supporting Information). This may contribute to the increase in the activity of the four proteins in the 1csc group. The stemness of the cells in the 1csc group is also indicated by the increased expression of two other commonly used protein markers of stemness, ALDHA1 and alkaline phosphatase (AP), compared with the other three groups (Figure 2e and Figure S12, Supporting Information). The capability of CSCs to survive in stressful conditions is correlated to high expression of anti‐apoptotic markers as well as protection of genome integrity by prompt activation of DNA damage sensor and repair machinery.^[^ ^18^ ^]^ Indeed, many DNA repair‐related genes including the *CCDH*, *ERCC*, *POLR2*, and *RPA* families are upregulated in the 1csc group compared with the other three groups (Figure 2d). In addition, the expressions of anti‐apoptotic genes (e.g., the *BCL2L2* and *CAP* families) are significantly higher in the 1csc group than the other three groups (Figure 2d). Cells in the 1csc group also express elevated levels of drug‐transporter proteins (e.g., the *ABCA1*, *ABCA8*, and *HSP90* families) to expel cytotoxic agents (Figure 2d), which may lead to high resistance to chemotherapeutic drugs. CSC quiescence has also been hypothesized to protect the cells against cytotoxic therapy.^[^ ^19^ ^]^ Indeed, many proliferation genes are downregulated in the 1csc group (e.g., *ABL1*, *FOXO1*, and *FOXO3*) compared with the other three groups (Figure 2d).

### 2.3 Cross‐tissue multilineage differentiation

**Figure 3:**
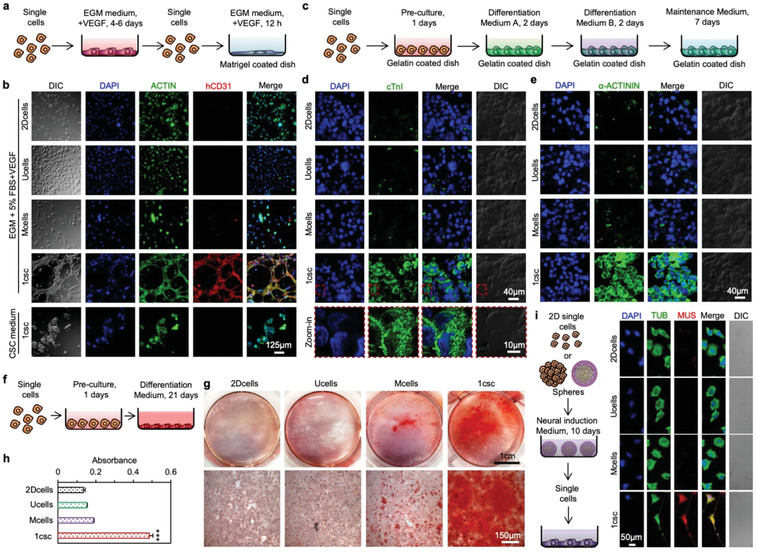
Characterization of stemness with cross‐tissue multilineage differentiation. a) A schematic illustration of the protocol for endothelial differentiation and vascular tube formation. b) Immunofluorescence staining of human CD31 (hCD31) and ACTIN and DAPI staining of nuclei in cells of the 2Dcells, Ucells, Mcells, and 1csc groups cultured in endothelial growth medium (EGM) supplemented with 50 ng mL^−1^ of vascular endothelial growth factor (VEGF), together with cells from the 1csc group cultured in CSC medium. DIC: Differential interference contrast. c) A schematic illustration of the protocol for cardiac differentiation. d,e) Confocal images showing the expression level of two cardiac specific markers, cardiac troponin I (cTnI, (d)) and *α*‐ACTININ (e), in cells of the 2Dcells, Ucells, Mcells, and 1csc groups after cardiac differentiation. f) A schematic illustration of the protocol for osteogenic differentiation. g) Typical pictures and microscopic images of the cells stained with Alizarin Red S to visualize calcium deposition after osteogenic differentiation. h) Quantitative analysis of the absorbance of Alizarin Red S at 500 nm in the cells after osteogenic differentiation from the 2Dcells, Ucells, Mcells, and 1csc groups. The *p* value for the 1csc group versus 2Dcells, Ucells, and Mcells groups is <0.0001. Statistical significance was assessed by one‐way ANOVA with post hoc Tukey test. ***p < 0.001. i) A schematic illustration of the protocol for neural differentiation together with confocal images showing the expression level of the neuron specific marker MUSASHI‐1 (MUS) and the formation of the neurites in the differentiated cells from the four groups. DAPI is for staining the cell nuclei and TUBULIN (TUB) is stained to show the cytoskeleton in cells. Error bars denote mean ± s.d.

Besides the expression of stemness genes and proteins, a crucial characteristic of stem cells is their capability of cross‐tissue multilineage differentiation.^[^ ^20^ ^]^ The MDA‐MB‐231 cells cultured with the aforementioned four methods were then investigated for their capacity of endothelial, cardiac, osteogenic, and neural differentiation. For endothelial differentiation, the cell colonies/spheroids/aggregates were dissociated and the 2D cultured cells were detached into single cells for culture in endothelial growth medium (EGM) supplemented with 50 ng mL^−1^ vascular endothelial growth factor (VEGF) for 4–6 days. Immunostainings of human CD31 and VE‐CADHERIN indicate that the cells derived from the 1csc group express significantly higher levels of the two endothelial cell markers compared with cells derived from the other three groups (Figure S13, Supporting Information). The expression of endothelial cell markers is minimal when the cells obtained from the 1csc colonies are cultured in the CSC medium, while the expression of OCT4 is stronger in the 1csc colony cells cultured in the CSC medium than the differentiation medium (Figure S13, Supporting Information). Moreover, the cells differentiated from the 1csc group are able to self‐assemble into blood vessel‐like structures after seeded on Matrigel in differentiation medium for 12 h (**Figure** **3**a,b and Figure S14, Supporting Information). These data indicate functional endothelial cells can be differentiated from the 1csc colony cells. In stark contrast, no blood vessel formation is observable when culturing the cells from the 2Dcells, Ucells, and Mcells groups on Matrigel in endothelial differentiation medium or the 1csc colony cells in CSC medium on Matrigel (Figure S14, Supporting Information). It is worth noting that cells from the 2Dcells, Ucells, and Mcells groups could attach and proliferate efficiently in culture medium or differentiation medium at least for three passages (Figure S15, Supporting Information). In contrast, the 1csc colony cells tend to form cell aggregates and most of them do not attach on the cell culture plate during passage 1. Surprisingly, starting from passage 2, the differentiated cells in the 1csc group could not proliferate and further passaging causes the cells to die and few cells could survive to passage 3 (Figure S15, Supporting Information). These data suggest the 1csc colony cells lose their cancerous property after endothelial differentiation and using VEGF to induce CSC endothelial differentiation might be a valuable strategy for cancer therapy. This may also contribute to the observation that VEGF inhibitor‐based cancer therapy is at times associated with cancer drug resistance.^[^ ^21^ ^]^

We further conducted cardiac differentiation on the cells obtained from the four different cultures using a two‐step differentiation assay from Thermo Fisher according to the manufacturer's instructions (Figure 3c and Figure S16, Supporting Information). Although we did not observe spontaneously beating cardiomyocytes, the immunostaining data show that high expression of multiple cardiac specific markers including cardiac troponin I (cTnI) and *α*‐ACTININ can be observed only for the 1csc group (Figure 3d,e and Figure S17, Supporting Information). Osteogenic differentiation was also conducted by utilizing a kit from Thermo Fisher as per the manufacturer's instructions (Figure 3f and Figure S18, Supporting Information) and staining of calcium deposits with Alizarin Red S was used to judge successful osteogenic differentiation.^[^ ^22^ ^]^ As shown in Figure 3g,h, significantly more calcium deposits could be observed in the 1csc group than the other three groups. Similarly, neural marker (MUSASHI‐1 or MUS staining) and neurite‐like structure could be observed only for the 1csc group after neural differentiation (Figure 3i and Figure S19, Supporting Information). All the aforementioned differentiation studies show that only the 1csc colony cells possess the capability of cross‐tissue multilineage differentiation, indicating they are truly CSCs. Cells obtained from the conventional Mcells, Ucells, and 2Dcells cultures may not be CSCs because they lack the key feature of cross‐tissue multilineage differentiation for stem cells.

### 2.4 In vivo tumorigenesis

**Figure 4:**
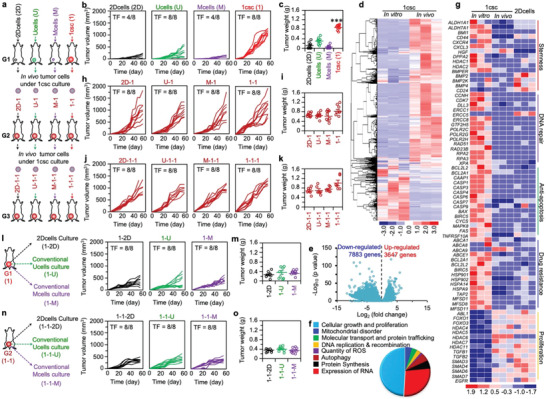
Characterization of stemness with multi‐generation tumorigenesis assay in vivo. a) A schematic illustration of the three‐generation (G1–G3) in vivo tumorigenesis assay. The G1 tumors were grown from cells obtained by 2Dcells, Ucells, Mcells, and 1csc cultures of the MDA‐MB‐231 cells; the G2 tumors were grown from cells obtained by 1csc culture of the four types of G1 tumor cells; and the G3 tumors were grown from cells obtained by 1csc culture of the G2 tumor cells in the four groups. b,c) Individual growth curves of the G1 tumors (2D, U, M, and 1) in 55 days (b) and weight of the G1 tumors on day 55 (c) for the four groups. The *p* value for the 1csc (1) group versus the 2Dcells (2D), Ucells (U), and Mcells (M) groups is <0.0001. Statistical significance was assessed by one‐way ANOVA with post hoc Tukey test. ***p < 0.001. d) Differential gene expression heat map from RNA‐Seq analysis of cells in the 1csc group before injection into mice (in vitro) and cells in tumor (in vivo) grown from the 1csc in vitro cells. e) The distribution of down‐ and upregulated genes in the in vivo cells as compared to the in vitro cells in the 1csc group. f) Pathway analysis of significantly altered genes in the 1csc in vivo cells as compared to the 1csc in vitro cells. The whole‐genome data are representative of three independent experiments. g) Heat map of gene expression in the 1csc in vitro and in vivo cells together with in vitro 2D cultured cells (2Dcells) regarding stemness (positive and negative), DNA repair, anti‐apoptosis, drug resistance, and cell proliferation. The gene expression of 1csc in vivo cells is closer to that of cells in the 2Dcells group than 1csc in vitro cells. h,i) Individual growth curves of G2 tumors (2D‐1, U‐1, M‐1, and 1‐1) in 55 days (h) and weight of the G2 tumors on day 55 (i) for the four groups. j,k) Individual growth curves of G3 tumors (2D‐1‐1, U‐1‐1, M‐1‐1, and 1‐1‐1) in 55 days (j) and weight of the G3 tumors on day 55 (k) for the four groups. l) A schematic illustration of the second‐generation tumors grown from cells obtained by 2Dcells, Ucells, and Mcells cultures of the 1csc G1 tumor cells (left), together with the individual growth curves of the tumors (1‐2D, 1‐U, and 1‐M) (right). m) Weight of the 1‐2D, 1‐U, and 1‐M tumors on day 55. n) A schematic illustration of the third‐generation tumors grown from cells obtained by 2Dcells, Ucells, and Mcells cultures of the 1csc G2 tumor cells (left), together with the individual growth curves of the tumors (1‐1‐2D, 1‐1‐U, and 1‐1‐M) (right). o) Weight of the 1‐1‐2D, 1‐1‐U, and 1‐1‐M tumors on day 55. TF: tumor formation in mice on day 55.

After confirming the stemness of the 1csc colony cells in vitro, we investigated the in vivo tumorigenic capability of the colony cells as compared to cells from the 2Dcells, Ucells, and Mcells groups. As schematically illustrated in **Figure** **4**a, three generations (G1–G3) of tumors were produced: the first generation (G1) tumors were generated using cells obtained from the MDA‐MB‐231 cells under 2Dcells (2D), Ucells (U), Mcells (M), and 1csc (1) cultures by injecting them into the mammary fat pads of immunodeficient mice; cells isolated from the G1 tumors (2D, U, M, and 1) of the four groups were then under the 1csc culture to obtain colony cells for injecting into the fat pads of immunodeficient mice to generate the second generation (G2) tumors (2D‐1, U‐1, M‐1, and 1‐1); and cells were again isolated from the G2 tumors and further cultured using the 1csc method to obtain colony cells for injecting into the fat pads of immunodeficient mice to generate the third generation (G3) tumors (2D‐1‐1, U‐1‐1, M‐1‐1, and 1‐1‐1). A total of 500 cells were injected into each mouse and the tumor growth was monitored for 55 days. Due to the small number of cells used for injection into each mouse, G1 tumor formation was observed in only 4 out of the 8 mice (TF = 4/8) for the 2Dcells and Mcells groups (Figure 4b,c and Figure S20a, Supporting Information). Although the G1 tumor formation does occur in 8 out of 8 mice (TF = 8/8) for both the Ucells and 1csc groups, the G1 tumors in the 1csc group grow much faster and are much bigger than that in all the other three groups. Interestingly, we do not observe any significant difference in tumor growth among the Ucells, Mcells, and 2Dcells groups, indicating the inability of the conventional methods for isolating CSCs (at least for the MDA‐MB‐231 cells line). To check the difference in transcriptome profile of the 1csc colony cells before (in vitro) versus after (in vivo) injection into mice, we performed RNA‐Seq on cells isolated from the G1 tumors in the 1csc group. As shown in Figure S20b (Supporting Information), all the triplicates correlate very well. Interestingly, many genes were differentially expressed between the in vitro 1csc colony cells and the cells of the in vivo tumors grown from the in vitro 1csc colony cells, as shown in the clustergram of the top 11,530 genes (Figure 4d and Figure S20c, Supporting Information). Specifically, 7883 genes are significantly downregulated whereas 3647 genes are significantly upregulated after injecting the in vitro 1csc colony cells into mice to grow in vivo tumors (Figure 4e). We further found that almost half of the upregulated genes in in vivo tumors were related to cellular growth and proliferation, suggesting that cellular proliferation of the 1csc colony cells is significantly altered after in vivo injection probably due to their spontaneous differentiation into tumor cells in vivo (Figure 4f). Additionally, the expression of most of the genes related to stemness, DNA repair, anti‐apoptosis, and drug resistance is significantly decreased in in vivo tumors compared to the in vitro 1csc colony cells and is similar to that in the 2D cultured cells (2Dcells, Figure 4g).

In order to examine if the 1csc culture approach can be used to isolate CSCs from in vivo tumors, cells from the G1 in vivo tumors of the four different groups (2D, U, M, and 1) were isolated and cultured using the 1csc approach with one single cell in each ACA@AH microcapsule in CSC medium. Interestingly, only ∼3–5% of the tumor cells are able to form colonies for all the four groups (Figure S21a, Supporting Information). Furthermore, all the 1csc colony cells obtained from the four groups are able to form the G2 (2D‐1, U‐1, M‐1, and 1‐1) in vivo tumors in the mouse mammary fat pads efficiently (TF = 8/8, Figure 4h,i and Figure S22a,b, Supporting Information). Similarly, the G3 in vivo tumor formation was performed by culturing cells from the G2 (2D‐1, U‐1, M‐1, and 1‐1) tumors using the 1csc culture to obtain colony cells for injecting into the mouse fat pads. As with cells from the G1 tumors, only ∼3–5% of the cells from the G2 tumors could form a colony in each ACA@AH microcapsule (Figure S21b, Supporting Information). Growth of the G3 (2D‐1‐1, U‐1‐1, M‐1‐1, and 1‐1‐1) tumors (Figure 4j,k and Figure S22c,d, Supporting Information) is similar to that of the aforementioned G2 and 1csc G1 tumors. Taken together, a total of three generations of in vivo tumorigenesis studies indicate that the colony cells from the 1csc culture are significantly much more tumorigenic than cells derived from the 2Dcells, Mcells, and Ucells cultures. To further confirm this, cells were isolated from the G1 (1) and G2 (1‐1) tumors grown from the 1csc colony cells and cultured using the three conventional culture methods (2Dcells or 2D, Ucells or U, and Mcells or M). The resultant cells were injected into the mouse fat pad for generating the second‐generation (1‐2D, 1‐U, 1‐M, Figure 4l,m) and third‐generation (1‐1‐2D, 1‐1‐U, and 1‐1‐M, Figure 4n,o) tumors. As shown in Figure 4l,m, the growth of the second‐generation 1‐2D, 1‐U, and 1‐M tumors is much slower than the G2 2D‐1, U‐1, M‐1, and 1‐1 tumors and the difference in tumor weight is significant (Figure S22a,b, Supporting Information). Similarly, the third‐generation 1‐1‐2D, 1‐1‐U, and 1‐1‐M tumors grow much slower than the G3 2D‐1‐1, U‐1‐1, M‐1‐1, and 1‐1‐1 tumors, and the difference in tumor weight is significant (Figure 4n,o and Figure S22c,d, Supporting Information).

To find out why the 1csc colony cells are highly tumorigenic, histology of the tumor tissues was analyzed through hematoxylin and eosin (H&E) staining. Interestingly, the percentage of necrotic area in the tumors grown from cells obtained with the 2Dcells, Ucells, and Mcells cultures is significantly larger than that in tumors grown from the 1csc colony cells for all three generations (G1–G3) (Figures S23–S25, Supporting Information). This is possibly because the 1csc colony cells can better regenerate the tumor microenvironment than cells obtained with the other three culture methods. For example, more blood vessels can be observed in the tumors grown from the 1csc colony cells than cells obtained with the other three culture methods (Figures S23–S25, Supporting Information), which may be attributed to the endothelial differentiation capability of the 1csc colony cells (Figure 3a,b). This is confirmed by immunofluorescence staining of human endothelial cell markers hCD31 and hVE‐CADHERIN: much higher expression of hCD31 and hVE‐CADHERIN can be observed in the 2D‐1‐1, U‐1‐1, M‐1‐1, and 1‐1‐1 tumors than 1‐1‐2D, 1‐1‐U, and 1‐1‐M tumors, although the expression of mouse endothelial cells marker (mCD31) is evident in all the tumors (Figure S26, Supporting Information). Increased blood vessels facilitate the transport of nutrients and oxygen inside tumors, which may contribute to the reduced necrosis observed in the tumors grown from the 1csc colony cells. It is worth noting that other mechanisms may contribute to the fast proliferation of tumors grown from the 1csc colony cells, such as the high expression of proliferating cell nuclear antigen (PCNA, Figure S27, Supporting Information) that promotes cell proliferation.

### 2.5 In vivo metastasis

**Figure 5:**
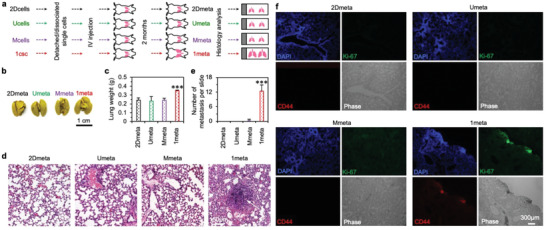
Characterization of stemness with metastasis assay in vivo. a) A schematic illustration of the protocol of the metastasis assay. Cells from the 2Dcells, Ucells, Mcells, and 1csc cultures are used for injection into mice via their tail vein and the corresponding metastasis study groups are called 2Dmeta, Umeta, Mmeta, and 1meta, respectively. b,c) Representative photographs of the Bouin solution‐fixed lungs (b) and the weight of the lungs (before fixation, (c)) from the 2Dmeta, Umeta, Mmeta, and 1meta groups. The *p* value for the 1meta group versus the 2Dmeta, Umeta, and Mmeta groups is 0.0005. Statistical significance was assessed by one‐way ANOVA with post hoc Tukey test. ***p < 0.001. d) Typical images of hematoxylin and eosin (H&E) stained lung tissue from the 2Dmeta, Umeta, Mmeta, and 1meta groups, showing metastasis (arrow) in the lungs of the 1meta group. e) Quantitative analysis of metastatic tumors observed in the H&E slide. The *p* values for the 1meta group versus the 2Dmeta, Umeta, and Mmeta groups is <0.0001 and statistical significance was assessed by one‐way ANOVA with post hoc Tukey test. ***p < 0.001. f) Representative fluorescence images of lung tissues from the 2Dmeta, Umeta, Mmeta, and 1meta groups after immunostaining for human CD44 and Ki‐67. The human CD44 and Ki‐67 could be seen only in the 1meta group. Error bars denote mean ± s.d.

Besides high tumorigenesis, CSCs have been posited to cause metastases. To investigate this, attached cells and cell aggregates/spheroids/colonies from the 2Dcells, Ucells, Mcells, or 1csc cultures were dissociated into single cells for injection into C57BL/6 mice with intact immune system via their tail veins (2 × 10^6^ cells per mouse, **Figure** **5**a). The mice were sacrificed after 2 months and the lungs from the four groups (2Dmeta, Umeta, Mmeta, or 1meta) were collected for further analyses. First, all the lungs in the 1meta group appear larger than that in the other three groups (Figure 5b), and all the lungs in the 1meta group are significantly heavier than that in all the other three groups (Figure 5c). This is possibly due to the formation of cancer metastases in this group. Indeed, further histology analyses with H&E staining show that metastases of a few hundred microns in size with densely packed cells formed in all the lungs of the 1meta group (Figure 5d,e). The metastatic tumors in the lungs of the 1meta group are further confirmed by immunofluorescence staining with human CD44 and Ki‐67 (Figure 5f). In stark contrast, no metastasis was observed in any of the lungs of the 2Dmet and Umeta groups and only one metastasis was observed in one of the lungs of the Mmeta group. This is probably because the cancer cells injected into mice for these groups are not stem cells and can be easily killed by the immune system of the mice. For the 1csc group, the colony cells are likely stem cells and may evade the immune system of the mice. The capability of human stem cells in evading the immune system to survive in murine species (mice and rats) has also been reported in the literature.^[^ ^23^ ^]^ It is worth noting that metastatic tumors were also observed in the liver and kidney of mice (2/8) in the 1meta group (Figure S28, Supporting Information). The seeding and growth of breast metastatic tumors at sites distant from the primary tumor is a complex and multistep process, and the epithelial‐mesenchymal transition (EMT) has been considered a major mechanism for breast cancer metastasis.^[^ ^24^ ^]^ Increased VIMENTIN expression is frequently used as an EMT marker in cancer and there is a positive correlation of VIMENTIN expression with augmented invasiveness and metastasis.^[^ ^25^ ^]^ Indeed, as shown in Figure S29 (Supporting Information), higher expression of VIMENTIN is observed in the 1csc colony cells than cells from the other three groups. Moreover, decreased expression of E‐CADHERIN and increased expression of *β*‐CATENIN are observed in the 1csc colony cells compared with cells in the other three groups (Figure S29, Supporting Information). Previous studies suggest that the suppression of E‐CADHERIN and increased expression of *β*‐CATENIN lead to mesenchymal phenotype, increased cell migration and invasion, and enhanced metastasis.^[^ ^26^ ^]^ Similarly, these data suggest that the 1csc colony cells possess stronger capacity of invasion and migration than cells in the other three groups.

### 2.6 Drug resistance and clinical significance

**Figure 6:**
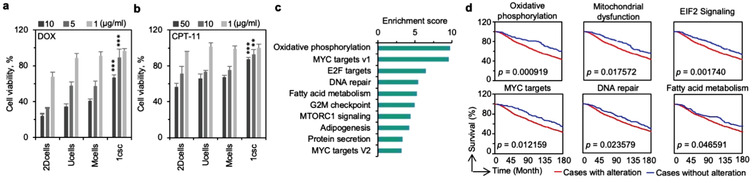
Implications for developing CSC‐targeted therapeutic strategies. a,b) Viability of the cells obtained by 2Dcells, Ucells, Mcells, and 1csc cultures of MDA‐MB‐231 cells after treating them with doxorubicin hydrochloride (DOX, (a)) and camptothecin‐11 (CPT‐11, (b)). For DOX, the *p* value for the 1csc group versus the 2Dcells, Ucells, and Mcells groups is <0.0001 for both 10 and 5 µg mL^−1^. For CPT‐11, the *p* value for the 1csc group versus the 2Dcells, Ucells, and Mcells groups is <0.0001 for 50 µg mL^−1^ and it is 0.0034 for 10 µg mL^−1^. Error bars denote mean ± s.d., and statistical significance was assessed by one‐way ANOVA with post hoc Tukey test. **p < 0.01, and ***p < 0.001. c) Gene set enrichment analysis (GSEA) of the signaling pathways enriched in 1csc colony cells compared with cells from the 2Dcells, Ucells, and Mcells cultures. d) Overall survival via Kaplan–Meier Estimate of patients with enriched core genes in oxidative phosphorylation, mitochondrial dysfunction, EIF2 signaling, MYC targets, DNA repair, and fatty acid metabolism pathways (red line). The blue line shows the survival of patients without enrichment in the respective pathways. Statistical significance was assessed by Kaplan–Meier survival analysis.

Having demonstrated the stemness of the 1csc colony cells through the serial passaging assay in vitro, gene and protein expression analyses of in vitro and in vivo cells, and studies on multilineage differentiation, three generations of in vivo tumorigenesis, and metastatic capacity, we next treated these cells with two chemotherapeutic drugs, doxorubicin hydrochloride (DOX) and camptothecin‐11 (CPT‐11). It is found that the 1csc colony cells are significantly more resistant to both drugs than cells obtained with the other three culture methods (**Figure** **6**a,b). This is not surprising, as the upregulation of many CSC‐related genes (e.g., DNA repair, anti‐apoptosis, and drug resistance) and downregulation of proliferation‐related genes (Figure 2d) should render the 1csc colony cells highly resistant to chemotherapeutic drugs. In order to find potential therapeutic targets of CSCs to overcome their drug resistance, differentially regulated canonical pathways as well as genes were analyzed. The results are shown in Figure S30 (Supporting Information). In conjunction to alterations in stemness related pathways (Figure S30a, Supporting Information), pathways related to energy metabolism (e.g., oxidative phosphorylation and mitochondrial dysfunction, Figure S30b, Supporting Information) are significantly altered in the 1csc group compared with the other three groups. Additionally, similar pathways (oxidative phosphorylation and mitochondrial dysfunction) are altered between the in vitro 1csc colony cells and in vivo tumors grown from them (Figure S31, Supporting Information), suggesting these pathways may play an important role in supporting/maintaining the stemness of the 1csc colony cells. Differences in energy metabolism are also revealed by utilizing gene set enrichment analysis (GSEA) of the top 23 enriched pathways with hallmarks (Figure S32, Supporting Information), and the top 10 are given in Figure 6c. These enriched pathways in the 1csc colony cells are involved in improved stemness, decreased cell growth, anti‐apoptosis, increased drug resistance, enhanced DNA repair, and reduced metabolism, which are considered important properties of CSCs (Figure 6c and Figure S32, Supporting Information). Most of the signaling pathways are also different between the in vitro 1csc colony cells and the in vivo 1csc tumor cells, and the top 39 are given in Figure S33 (Supporting Information).

Lastly, we examined the clinical significance of targeting the CSCs isolated with the 1csc culture by investigating the survival of breast cancer patients with alterations in the top enriched gene sets found from our GSEA analyses of the 1csc colony cells as compared to cells in the 2Dcells, Ucells, and Mcells groups. These include oxidative phosphorylation, mitochondrial dysfunction, EIF2 targets, MYC targets, DNA repair, and fatty acid metabolisms (Figure 6c and Figures S30–32, Supporting Information). As shown by the Kaplan Meier estimates of survival (Figure 6d), alterations of these gene sets decrease the prognostic outcomes in breast cancer patients, suggesting that they can be potential therapeutic targets for eliminating CSCs to improve breast patient survival. Perhaps, the combination of inhibitors of these pathways and/or their further combination with factors for guided differentiation (e.g., VEGF for endothelial differentiation so that the cells lose cancerous property and do not proliferate, Figure S15, Supporting Information) may be used to target CSCs and achieve improved outcomes of breast cancer therapy.

## 3 Conclusions

In conclusion, inspired by the prehatching embryos which start proliferation from one cell to form a colony, we developed a one‐single‐cell‐microencapsulation (1csc) method to isolate and culture CSCs without needing any surface marker. Hyaluronic acid was found to be crucial for the CSCs to survive and form colonies under the 1csc culture and the CSCs take up ∼3–5% the whole cancer cell population. Genome profiling indicates that the stemness, DNA repair, anti‐apoptosis, and drug resistance of the 1csc colony cells are enhanced while their tendency of proliferation is decreased compared with 2D cultured cells and cells obtained with the conventional approaches for isolating CSCs. Furthermore, the CSCs obtained with the 1csc culture are capable of cross‐tissue multilineage (e.g., endothelial, cardiac, osteogenic, and neural) differentiation. Moreover, three generations of in vivo tumorigenesis studies together with investigations on metastasis indicate that the CSCs are highly tumorigenic and metastatic. The CSCs are also shown to be highly resistant to chemotherapeutic drugs DOX and CPT‐11. Our one‐single‐cell‐microencapsulation approach for CSC isolation and culture may be valuable for understanding cancer biology and etiology and for facilitating the development of CSC‐targeted effective therapies to fight against cancer.

## Conflict of Interest

The authors declare no conflict of interest.

## Supporting information

Supporting Information including detailed information on materials and methods and the supporting figures and table
