# Bioinspired One Cell Culture Isolates Highly Tumorigenic and Metastatic Cancer Stem Cells Capable of Multilineage Differentiation

**DOI:** 10.1002/advs.202301726

**Published:** 2023-04-26

**Authors:** Hai Wang, Pranay Agarwal, Bin Jiang, Samantha Stewart, Xuanyou Liu, Yutong Liang, Baris Hancioglu, Amy Webb, John P. Fisher, Zhenguo Liu, Xiongbin Lu, Katherine H. R. Tkaczuk, Xiaoming He

*Adv. Sci*. **2020**, *7*, 2000259

https://doi.org/10.1002/advs.202000259

In the originally published article, some figures in the Supporting Information were incorrect. In these figures, incorrect images were used by accident for the control conditions of 1% alginate hydrogel of Day 5 in Figure S3a, Ucells in Figure S12b, Day 1 of 2D cells (Passage 3, EGM) in Figure S15, Ucells (Differentiation medium A) and Mcells (Maintenance medium) in Figure S16, and of 1‐1 and M‐1‐1 in Figure S27, which occurred during initial the preparation of the figures. The corrections do not affect the conclusions of the article. Supporting Information file was updated on April 26, 2023. The authors apologize for any inconvenience caused.

