# Supplementary material for: Bioinspired One Cell Culture Isolates Highly Tumorigenic and Metastatic Cancer Stem Cells Capable of Multilineage Differentiation
Source: Adv Sci (Weinh). 2020 Apr 28;7(11):2000259. doi: 10.1002/advs.202000259 (PMC7284220; doi:10.1002/advs.202000259)
Supplement: Supplementary file 1 — Supporting Information including detailed information on materials and methods and the supporting figures and table [file ADVS-7-2000259-s001.pdf]

Copyright WILEY-VCH Verlag GmbH & Co. KGaA, 69469 Weinheim, Germany, 2018.

Supporting Information

Bioinspired one cell culture isolates highly tumorigenic and metastatic cancer stem cells capable of multilineage differentiation

Hai Wang, Pranay Agarwal, Bin Jiang, Samantha Stewart, Xuanyou Liu, Yutong Liang, Baris Hancioglu, Amy Webb, John P. Fisher, Zhenguo Liu, Xiongbin Lu, Katherine H.R. Tkaczuk, Xiaoming He**

Materials and Methods

Materials. Chitosan oligosaccharide of pharmaceutical grade (MW: 80 kDa, 95.5% deacetylation) was purchased from Zhejiang Golden-Shell Biochemical Co. Ltd (Zhejiang, China). Hyaluronan (HA, MW: 151-300 kDa) was purchased from Lifecore Biomedical (Chaska, MN, USA). Doxorubicin hydrochloride (DOX) was purchased from LC laboratories (Woburn, MA, USA). Irinotecan/camptothecin-11 (CPT-11) was purchased from Selleck Chemicals (Houston, TX, USA). The cell counting kit-8 (CCK-8) cell proliferation reagent was purchased from Dojindo Molecular Technologies (Rockville, MD, USA). Fetal bovine serum (FBS) and penicillin/streptomycin were purchased from Invitrogen (Carlsbad, CA, USA). The DMEM, EMEM, F12K, RPMI-1640, and DMEM/F-12K cell culture media were purchased from ATCC (Manassas, VA, USA). Sodium alginate was purchased from Sigma (St. Louis, MO, USA) and further purified by washing in chloroform and charcoal and dialyzing (MWCO: 50 kD) for 24 h, followed by freeze-drying to remove water. All other chemicals were purchased from Sigma unless specifically mentioned otherwise.

Fabrication of microfluidic devices. Polydimethylsiloxane (PDMS) based microfluidic devices were fabricated as described previously.[1] Briefly, a non-planar silicon master with patterned microfluidic channels was prepared by utilizing a 3-layer SU8 fabrication technique. To do this, a 100 μm-thick layer of SU8 2050 was coated on a 4 -inch silicon wafer. The wafer was then soft-baked at 95 °C, followed by exposure to UV light through a shadow mask for the core channel. After post-exposure baking, an additional layer (50-μm thick) of SU8 2050 was coated and baked at 95 °C. Thereafter, the wafer was exposed to UV light with a different shadow mask to pattern the shell channel. Afterward, a third SU8 2050 layer of 50-μm thickness was coated and baked at 95 °C. Finally, the wafer was exposed to UV light with a shadow mask to pattern the oil and extraction channels. All three exposures were aligned by utilizing an EVG620 mask aligner. In the end, the SU8 pattern was developed in SU8 developer solution. To fabricate PDMS microfluidic devices, a PDMS pre-polymer solution (at 10:1 ratio of the pre-polymer to its curing agent) was poured onto the silicon wafer followed by baking at 65 °C for a minimum of 3 hours. Thereafter, two PDMS slabs with identical channel design were plasma treated for 30 s using the Harrick PDC-32G plasma cleaner and aligned under microscope to form an assembled device. The devices were kept at 65 °C for at least 2 days to make them sufficiently hydrophobic for further experimental use.

Microencapsulation of one single cell and ACA coating. The fluid in the core channel was an aqueous sodium alginate solution (2%) with or without 0.5% hyaluronic acid (HA), whereas the fluid in the shell channel was the 2% alginate solution. To encapsulate one single cell in each microcapsule, the core solution was suspended with cells at 10^4 cells ml^{-1}. Solution in the extraction channel (Figure S2) was composed of 0.5%wt high-viscosity sodium carboxymethyl cellulose and 0.5%wt low-viscosity sodium carboxymethyl cellulose. All solutions were prepared in 0.3 M D-Mannitol solution and buffered with 10 mM HEPES to maintain the neutral pH 7.4. In order to crosslink alginate in the microcapsules, stable emulsion composed of mineral oil and 1 g ml^{-1} aqueous calcium chloride solution (volume ratio: 10 to 3 with the addition of 1.5% SPAN 80)) was prepared by sonication for 1 min using a Branson 450 digital sonifier. All the solutions were injected into the microfluidic device using a syringe pump to generate microcapsules. Flow rates for core, shell, oil, and aqueous extracting fluids were 120 μl hr^{-1}, 220 μl hr^{-1}, 4 ml hr^{-1}, and 4ml hr^{-1}, respectively. Outlets were connected to a 50 ml tube containing the cell culture medium to collect the microcapsules. For ACA coating, a layer-by-layer coating approach was utilized.[2] Microcapsules after collection were washed twice with mannitol and suspended in a 0.4%wt chitosan (in 0.9% saline) solution for 30-40 seconds to form the AC coating. Thereafter, microcapsules were washed twice with isotonic mannitol solution to remove chitosan. The AC coated microcapsules were then suspended in an 0.2%wt sodium alginate (in 0.9% saline) solution for 2-3 minutes to form a stable alginate coating. Finally, the resultant microcapsules with ACA coating were washed twice with the mannitol solution and placed in growth medium for culture. Alginate is a negatively charged polymer which forms strong electrostatic

interactions with the positively charged chitosan polymer.[2] Lastly, the microcapsules contained two or more cells were removed by pipetting with a 100-μl pipette tip under microscope.

Cell culture and in vitro cell viability. For 2Dcells culture, human MDA-MB-231, MCF-7, PC-3, and OVCAR-8 cancer cells were cultured in Corning (Lowell, MA, USA) T75 flasks in DMEM, EMEM, F12K, and RPMI-1640 medium supplemented with 10% FBS and 1% penicillin/streptomycin at 37 °C in a humidified 5% CO_2 incubator, respectively. For Ucells culture, cells were cultured in Corning (Lowell, MA, USA) 6-well ultralow attachment plate (ULAP) at a density of 20,000 cells ml^{-1} in CSC medium consisting of serum-free DMEM/F12-K supplemented with 5 μg ml^{-1} insulin, 20 ng ml^{-1} epidermal growth factor (EGF), 20 ng ml^{-1} basic fibroblast growth factor (bFGF), 1x B27 (Invitrogen, Carlsbad, CA, USA), 0.4% (w v^{-1}) bovine serum albumin, 100 U ml^{-1} penicillin, and 100 μg ml^{-1} streptomycin. The cell spheroids or aggregates were collected after 7 days for further experiments. For 1csc and Mcells cultures, microcapsules encapsulated with one (for 1csc) or multiple (for Mcells) cells were cultured in the aforementioned CSC medium. To determine viability of cells under 2Dcells culture, cells were transferred into 96-well plates first. After 12 h, the pure medium was replaced with medium containing various drug formulations and further cultured for 24 h. For Ucells, Mcells, and 1csc cultures, cell aggregates/spheroid/colonies were divided equally into three groups and cultured with medium containing various drug formulations in 24-well plate for 24 h. The cell viability was then evaluated using the CCK-8 cell proliferation reagent per the manufacturer's instructions. Cell viability was calculated as the ratio of the cell number determined for each group with a treatment to that of control group with no treatment.

RNA sequencing. PicoPure RNA Isolation Kit was used to extract RNAs from cells in the 1csc group because of the small number of cells available in this group, while RNAs in cells from the other three groups were isolated with a Qiagen (Germantown, MD, USA) RNAeasy Plus Mini Kit. Quality and quantity of the extracted RNAs were analyzed using an Agilent Technology 2100 Bioanalyzer with a high sensitivity DNA chip (RIN > 8) (Table S1). RNAs were processed through SMARTer Ultra Low RNA Seq Kit v4 (Clontech) for library preparation according to the manufacturer's instructions. Libraries were finally sequenced on an Illumina HiSeq sequencer with paired (2 x 125 base pairs) end reads at the DNA Sequencing Center in Brigham Young University, Provo, UT, USA.

Differential gene expression analysis. RNA-sequencing quality was analyzed using the FASTQC program (http://www.bioinformatics.babraham.ac.uk/projects/fastqc/). Reads were trimmed and aligned to the GRCh38 human reference genome. At least 30 million reads per sample were aligned to the genome. Differential gene expression analyses were performed using DESEQ2. For our downstream analyses, we focused on transcripts with change greater than 2.0 in expression and adjusted p value less than 0.05. Pathway analyses on the differentially expressed genes were performed using the ingenuity pathway analysis (IPA, Qiagen) method. Gene set enrichment analysis (GSEA, http://www.broadinstitute.org/gsea) was performed to determine if the predefined gene sets (hallmark gene sets downloaded from the Molecular Signature Database available from Broad Institute, Cambridge, MA, USA) show significant differences between the 1csc and other groups.

In vitro imaging. For imaging OCT4, SOX2, NANOG, and KLF4, cells from the 2Dcells (cultured on collagen-coated cover glasses), Ucells, Mcells, and 1csc groups were fixed with 4% paraformaldehyde (PFA) for 20 min at room temperature. After washing with PBS for 3 times, the fixed cells were incubated in 0.1% TritonX-100 and 3% BSA in PBS at room temperature for 1 h to permeabilize the cell plasma membrane and block nonspecific binding. Then, the cells were incubated at 4 °C with OCT4 (Abcam, Cambridge, MA, USA, ab18976), SOX2 (Abcam, ab97959), NANOG (Abcam, ab109250), and KLF4 (Abcam, ab215036) antibodies at the dilution ratio of 1:200. After 12 h, the unbounded antibody was removed by washing with PBS for three times. Afterward, the cells were incubated with FITC-conjugated secondary antibody (Thermo Fisher) at the dilution ratio of 1:200 in PBS with 1% BSA at room temperature for 1 h, followed by washing for three times with PBS. The cells were then covered with cover glass and

anti-fade mounting medium (Vector Laboratories Burlingame, CA, USA) for examination using an Olympus FluoView™ FV1000 confocal microscope.

Flow cytometry. Samples of the Mcells and 1csc groups were incubated with an isotonic solution of sodium citrate first to release cell spheroids/colonies from the microcapsules by pipetting. These cell spheroids and colonies together with cells in the 2Dcells and Ucells groups were treated with 0.25% Trypsin to obtain detached single cells (3-5 × 10^5 cells per sample). The cells were then fixed, permeabilized, and blocked with non-specific binding in the same way as aforementioned for *in vitro* imaging. Afterward, the cells were incubated with antibodies at the dilution ratio of 1:200 at room temperature for 1 h and the unbounded antibody was removed by washing with PBS for three timesThe cells were then incubated with FITC-conjugated secondary antibody (Thermo Fisher) at the dilution ratio of 1:200 in PBS with 1% BSA at room temperature for 1 h, followed by washing for three times with PBS. Lastly, the cells were analyzed using a BD (Franklin Lakes, NJ, USA) LSR-II flow cytometer and Diva software. When needed, the cells were collected by centrifugation at 400 g for 5 min without significant cell loss during the procedure.

Endothelial differentiation. The endothelial differentiation was conducted by following a previously published protocol with slight modification.[3] Briefly, dissociated single cells of the four groups were obtained in the same way as aforementioned for flow cytometry studies and cultured with the endothelial growth medium (EGM) supplemented with 50 ng ml^{-1} VEGF in 6-well plate for 4-6 days. For tube formation, Matrigel (Corning, Lowell, MA, USA) was added into 24-well plates and incubated at 37°C for 30 min to coat the plates. The gels were then overlaid with 1 × 10^5 cells suspended in the EGM medium and incubated for 12 h. Successful endothelial differentiation was confirmed with immunostainings of human CD31 (Abcam, ab28364) and VE-cadherin (Cell Signaling Technology, Danvers, MA, USA, #2158) at the dilution ratio of 1:200 and 1:100, respectively. For the immunostaining, cells were fixed (without permeabilization) and blocked for non-specific binding in the same way as aforementioned for *in vitro* imaging. For actin staining, fixed cells were incubated with FITC-labeled phalloidin (Sigma, 10 μg ml^{-1} in PBS) for 30 min and washed with PBS for three times before imaging. The imaging was conducted in the same as that mentioned above.

Cardiac differentiation. Cardiac differentiation was conducted using the PSC Cardiac Differentiation Kit (Thermo Fisher, A2921201) according to the manufacturer's instructions. Briefly, dissociated single cells obtained as aforementioned were cultured in 6-well plate for 24 h in either DMEM medium for 2Dcells or CSC medium for other groups and then replaced with pre-warmed Cardiomyocyte Differentiation Medium A given in the kit. After 2 days, the medium was aspirated slowly from each well and replaced with pre-warmed Cardiomyocyte Differentiation Medium B given in the kit. Following culture for 2 days, the medium in each well was replaced with pre-warmed Cardiomyocyte Maintenance Medium given in the kit and the medium was changed every two days. The cells were then collected for further characterization after one week of culture. For immunostaining, cells were cultured on collagen-coated cover glass, blocked, fixed, and permeabilized as aforementioned. Cells were then incubated at 4 °C with cTnI (Abcam, ab47003) and α-ACTININ (Sigma, A7811) antibodies at the dilution ratio of 1:200. After 12 h, the unbounded antibody was removed by washing with PBS for three times. Cells were then incubated with secondary antibody at the dilution ratio of 1:200 in PBS with 1% BSA at room temperature for 1 h and washed for three times with PBS. Afterward, the cells were covered with cover glass and anti-fade mounting medium (Vector Laboratories Burlingame, CA, USA) for examination using an Olympus FluoView™ FV1000 confocal microscope. For flow cytometry studies, the cells after cardiac differentiation were detached by trypsin and stained with cTnI and α-actinin in the same way as aforementioned and further analyzed using a BD (Franklin Lakes, NJ, USA) LSR-II flow cytometer and Diva software.

Osteogenic differentiation. This was conducted using the StemPro™ Osteogenesis Differentiation Kit (Thermo Fisher, A1007201) according to the manufacturer's instructions. Briefly, single cells obtained as aforementioned were cultured in 6-well plate for 24 h in either DMEM medium for 2Dcells or CSC medium

for other groups. Afterward, the medium was slowly replaced with pre-warmed Osteogenesis Differentiation Medium given in the kit. After 21 days of culture, the cells were stained with Alizarin Red S given in the kit to stain and visualize calcium deposition in the samples.

Neural differentiation. The neural induction was done by following a previously published protocol with slight modification.[4] Briefly, single cells obtained as aforementioned for all the four groups were plated on a cell culture dish and cultured to 50-70% confluency in either DMEM medium for the 2Dcells group or CSC medium for other groups. Then, the culture medium was replaced with neural induction medium prepared by supplementing the neural basal medium (Gibco, Gaithersburg, MD, USA) with 10 μM SB431542 (Sigma) and 1 μM desomorphine (Sigma), 1x N2 (Gibco), 1x B27 (Gibco), and 1mM L-glutamine. The cells were cultured for 10 days with the medium being changed every other day. For immunostaining, cells were processed as aforementioned with primary antibodies of MUSASHI-1 and β-TUBULIN (R&D Systems, Minneapolis, MN, USA) at the dilution ratio of 1:500. The nuclei were stained with 1 μM DAPI at room temperature for 5 min. Images were taken using a Zeiss (Thornwood, NY, USA) LSM 710 confocal scanning microscope.

Animals and animal experiments. All animal experiments were performed in accordance with the "Guide for the Care and Use of Laboratory Animals of the US National Institutes of Health". The experimental protocols were reviewed and approved by the Institutional Animal Care and Use Committee of the Ohio State University and all efforts were made to minimize animal suffering. Both the female NOD SCID and C57BL/6 mice of 6-week old were purchased from Charles River (Wilmington, MA, USA) and maintained on a 16:8 h light-dark cycle.

To investigate tumorigenesis, detached single cells were suspended at 5,000 cells ml^{-1} in a mixture (1:1) of PBS and Matrigel. A total of 500 cells in 100 μl of the mixture was injected into the fat pad of each 7-week-old female NOD SCID mouse. Tumor growth was monitored every 5 days. The tumor volume was calculated as: $V = (L \times W^2) \times 0.5$, where L is long diameter and W is short diameter of the tumors determined using a caliper. The mice were euthanized on day 55 and tumors were collected. For histological analyses, tumors were fixed with 4% PFA, embedded with paraffin, and stained with hematoxylin and eosin (H&E).

For 2Dcells, Ucells, Mcells, or 1csc culture of *in vivo* tumor cells, fresh tumors were gashed into small squares (~0.5 mm^3) and put in a 50 ml conical tube. After washed with Hank's balanced salt solution (HBSS, Thermo Fisher) containing calcium and magnesium for 3 times, the small squares were incubated with collagenase (100 U ml^{-1}, Life Technologies, NY, USA) for 4 h at 37 °C with careful agitation of the samples approximately every half an hour. The dispersed cells were collected into another sterile 50-ml conical tube by passing through filtration with a 40-μm sterile nylon mesh cell strainer. The cells were then collected by centrifugation at 200 g for 5 min and further treated with 0.25% Trypsin to obtain dissociated single cells. The dissociated cells were cultured in Corning (Lowell, MA, USA) T75 flasks in DMEM medium for 12 h and then replaced with fresh medium to remove dead cells and debris. After cultured to 70-80% confluency, the cells were detached with 0.25% Trypsin and further cultured in ultralow attachment plate for 3 days with DMEM medium to remove the fibroblasts. Finally, the tumor cells were collected by centrifugation at 200 g for 5 min and treated with 0.25% Trypsin for 2Dcells, Ucells, Mcells, or 1csc culture as aforementioned in the microencapsulation or *in vitro* cell culture sections.

To investigate metastasis, dissociated single cells obtained as aforementioned for the four groups were intravenously injected into the C57BL/6 mice of 7-week old through the tail vein (2×10^6 cells per mouse and 8 mice per group). After 2 months, mice were sacrificed, and their major organs collected. The lungs were fixed with Bouin solution (Sigma) for imaging. For immunostaining of CD44 (Abcam, ab157107) and Ki-67 (Thermo Fisher, MA5-14520), lungs were collected and then frozen with the Tissue-Tek (Sakura Finetek, Torrance, CA, USA) O.C.T. Compound and Cryomold at -80 °C for 24 h. The lungs were then cut

into slices of 10-μm thick using a cryo-microtome (Leica Biosystems Inc, IL, USA) and transferred onto microscope slides. The immunostaining was conducted as aforementioned at the dilution ratio of 1:200 for both antibodies. For histological analysis, lungs, kidneys, and livers were fixed with 4% PFA, embedded with paraffin, and then stained with H&E as aforementioned.

Immunostaining of tumor tissue. For staining of mouse CD31 (mCD31, R&D Systems, AF3628), human CD31 (hCD31, R&D Systems, BBA7), and human VE-cadherin (hVE-cadherin, Cell Signaling Technology, #2158), tumors collected at day 55 were conducted as aforementioned to obtain the 10 μm-thick slices for immunostaining. The slides were incubated in 3% BSA and 0.1% TritonX-100 in 1x PBS at room temperature for 1 h, followed by overnight incubation at 4 °C with mouse CD31 and hVE-cadherin antibodies. The samples were then washed for 3 times with PBS and incubated in the dark at room temperature for 1 h with Alexa 680 and Rhodamine B-labeled secondary antibody (Thermo Fisher) diluted (1:50 dilution) in 1x PBS containing 1% BSA. Afterward, the preparations were incubated with hCD31 antibody at 4 °C overnight, washed for 3 times with PBS, and incubated with FITC-labeled secondary antibody (Thermo Fisher, diluted at 1:50 dilution in 1x PBS containing 1% BSA) in the dark at room temperature for 1 h. For PCNA staining, the tumor tissues on glass slides were incubated in PCNA antibody (Sigma, AV03018) at 4 °C for 12 h after blocking potential non-specific binding and permeabilizing with 3% BSA and 0.1% TritonX-100 in 1x PBS at room temperature for 1 h. The slides were then washed for 3 times with PBS and incubated in dark at room temperature for 1 h with FITC-labeled secondary antibody (Abcam) diluted in PBS containing 1% BSA. Finally, the preparations were washed and further stained for nuclei using Hoechst 33342 for examination with an Olympus FV1000 confocal microscope.

EMT staining. Attached 2D cells (for 2Dcells culture), spheroids (for Mcells and Ucells cultures), and colonies (for 1csc culture) were fixed with 4% paraformaldehyde for 20 min at room temperature. All samples were then washed for 3 times with PBS and incubated in 3% BSA and 0.1% TritonX-100 in PBS at room temperature for 1 h to block nonspecific binding and permeabilize the cell plasma membrane, respectively. Afterwards, samples were incubated overnight with primary antibodies (Cell Signaling Technology, EMT antibody sampler kit # 9782) including VIMENTIN (1:100), E-CADHERIN (1:200), and β-CATENIN (1:100). Next day, unbounded antibody was removed by washing with PBS for three times. Samples were then incubated with FITC-labeled secondary antibody at the dilution ratio of 1:200 in PBS with 1% BSA at room temperature for 1 h and washed for three times with PBS. Finally, samples were mounted using an antifade mounting medium (Vector Laboratories Burlingame, CA, USA) for examination using an Olympus FluoView™ FV1000 confocal microscope.

Statistical analysis. All data are reported as mean ± standard deviation (s.d.) from at least three independent runs. One-way ANOVA with post hoc Tukey test was used for comparison among more than two groups by GraphPad Prism8 Software. For the patient survival analysis, Kaplan-Meier method was used and carried out with the IBM SPSS 22 software. The invasive breast cancer dataset[5] from the cBioPortal for Cancer Genomics database was used for the patient survival analysis. In all cases, a p value less than 0.05 was considered to be statistically significant.

Data availability. All data supporting the findings of this study are available from the corresponding authors upon request.

References

[1] P. Agarwal, J. K. Choi, H. Huang, S. Zhao, J. Dumbleton, J. Li, X. He, *Part. Part. Syst. Charact.* **2015**, *32*, 809; P. Agarwal, S. Zhao, P. Bielecki, W. Rao, J. K. Choi, Y. Zhao, J. Yu, W. Zhang, X. He, *Lab Chip* **2013**, *13*, 4525.

[2] W. Zhang, S. Zhao, W. Rao, J. Snyder, J. K. Choi, J. Wang, I. A. Khan, N. B. Saleh, P. J. Mohler, J. Yu, T. J. Hund, C. Tang, X. He, *J. Mater. Chem B* **2013**, *1*, 1002; S. Zhao, L. Zhang, J. Han, J. Chu, H.

Wang, X. Chen, Y. Wang, N. Tun, L. Lu, X. F. Bai, M. Yearsley, S. Devine, X. He, J. Yu, *ACS Nano* **2016**, *10*, 6189.

[3] L. Vitiani, R. Pallini, M. Biffoni, M. Todaro, G. Invernici, T. Cenci, G. Maira, E. Parati, G. Stassi, L. Larocca, R. Maria. Tumour vascularization via endothelial differentiation of glioblastoma stem-like cells. *Nature* **2010**, *468*, 824.

[4] A. Morizane, D. Doi, T. Kikuchi, K. Nishimura, J. Takahashi, *J. Neurosci. Res.* **2011**, *89*, 117; S. M. Chambers, C. A. Fasano, E. P. Papapetrou, M. Tomishima, M. Sadelain, L. Studer, *Nat. Biotechnol.* **2009**, *27*, 275.

[5] B. Pereira, S. F. Chin, O. M. Rueda, H. K. Vollan, E. Provenzano, H. A. Bardwell, M. Pugh, L. Jones, R. Russell, S. J. Sammut, D. W. Tsui, B. Liu, S. J. Dawson, J. Abraham, H. Northen, J. F. Peden, A. Mukherjee, G. Turashvili, A. R. Green, S. McKinney, A. Oloumi, S. Shah, N. Rosenfeld, L. Murphy, D. R. Bentley, I. O. Ellis, A. Purushotham, S. E. Pinder, A. L. Borresen-Dale, H. M. Earl, P. D. Pharoah, M. T. Ross, S. Aparicio, C. Caldas, *Nat. Commun.* **2016**, *7*, 11479.

Supporting Figures

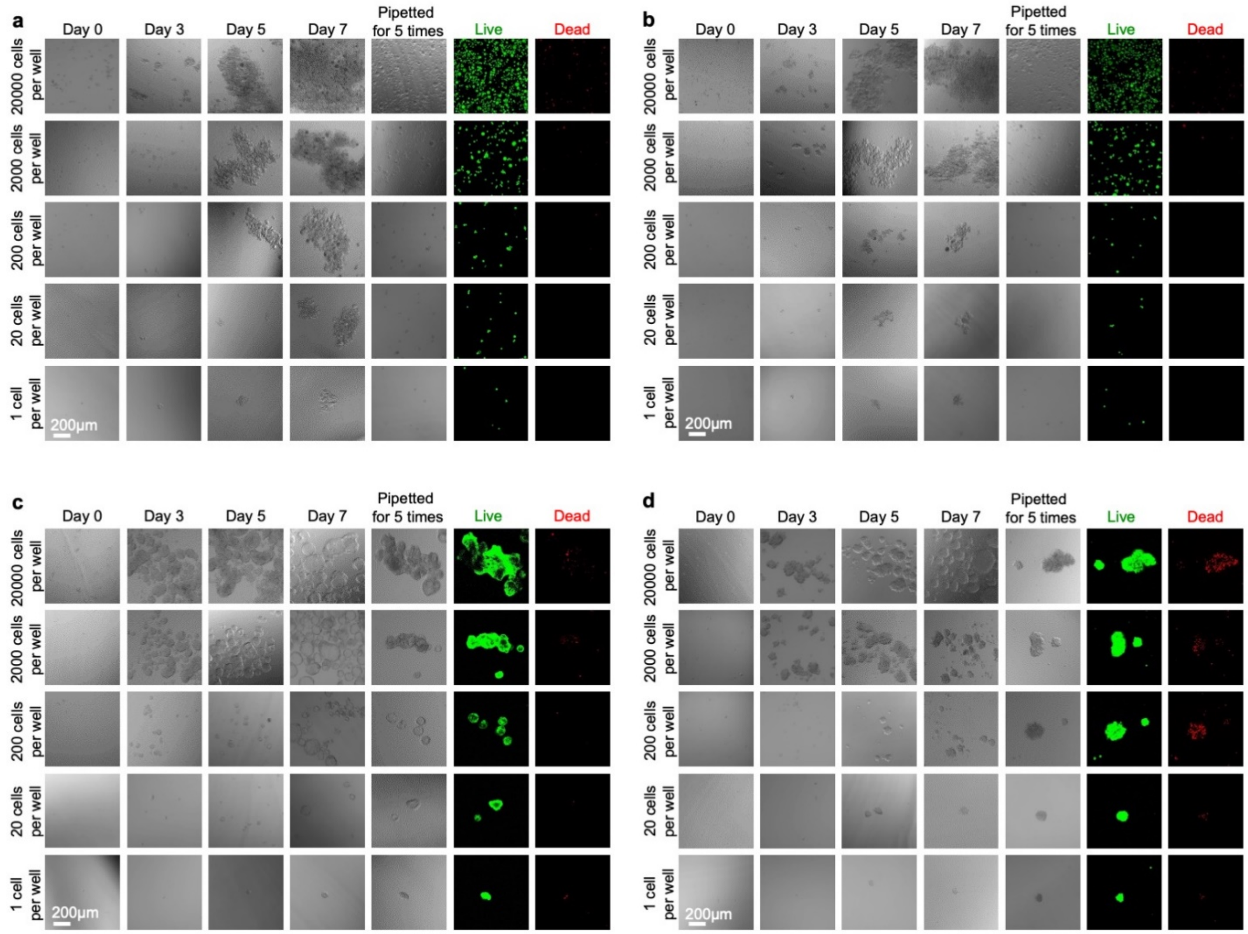

Figure S1. Capability of aggregate formation of four different types of cancer cells cultured in ultralow attachment plate (ULAP). Typical micrographs of MDA-MB-231 triple negative human breast cancer cells (**a**), PC-3 human prostate cancer cells (**b**), MCF-7 HER2$^+$ human breast cancer cells (**c**), and OVCAR-8 human ovarian cancer cells (**d**) cultured in ULAP with different cell densities show most of the cells could form aggregates. The MDA-MB-231 and PC-3 aggregates can be easily detached/dissociated into single cells by pipetting for five times, but the MCF-7 and OVCAR-8 aggregates do not detach/dissociate into single cells after five times of pipetting. Live/dead (green/red) staining shows most of the cells are alive after the gentle pipetting.

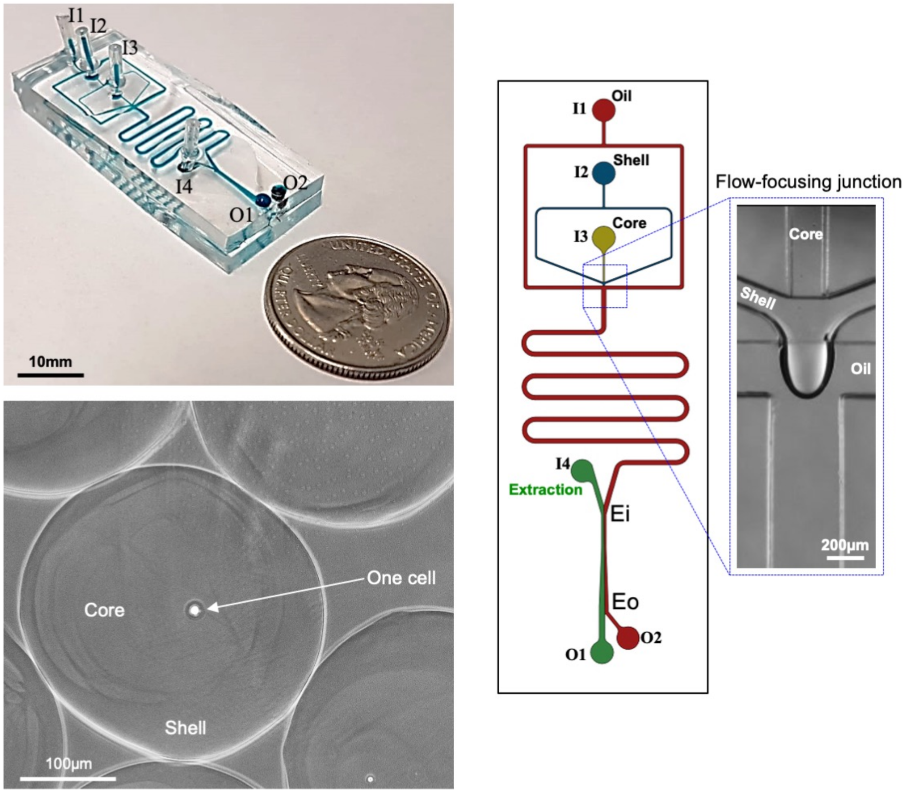

Microchannel	Dimension, μm	
Core	Height: 200<br>Width: 200	
Shell	Height: 300<br>Width: 300	
Oil	Height: 400<br>Width: 200	
Extraction	At Ei	Height: 400<br>Width: 500
	At Eo	Height: 400<br>Width: 800

Figure S2. The microfluidic device (top left: real image and middle: a schematic diagram and real image) used for one single cell microencapsulation with the flow-focusing mechanism in this study. The microchannel system in the real image of the device on the top left is filled with the aqueous solution of 0.08% (w v^{-1}) Fast Green FCF dye (Sigma) for better visualization of the microchannels. The bright field image in the middle shows the design of the flow-focusing junction and the table on the right indicates the dimensions of the various channels in the device. The height is in the direction perpendicular to the schematic diagram in the middle. Mineral oil emulsified with aqueous calcium chloride solution, aqueous sodium alginate solution (to form the microcapsule shell), a mixture of aqueous sodium alginate and hyaluronan (HA) solutions (with or without cells) to form the microcapsule core, and aqueous extraction solution are pumped into the device via inlets I1, I2, I3, and I4, respectively. The aqueous extraction solution (containing core-shell microcapsules) and mineral oil exit the device from outlets O1 and O2, respectively. Also shown is a typical image (bottom left) of the one cell-laden microcapsule with a core-shell configuration collected from the aqueous exit O1.

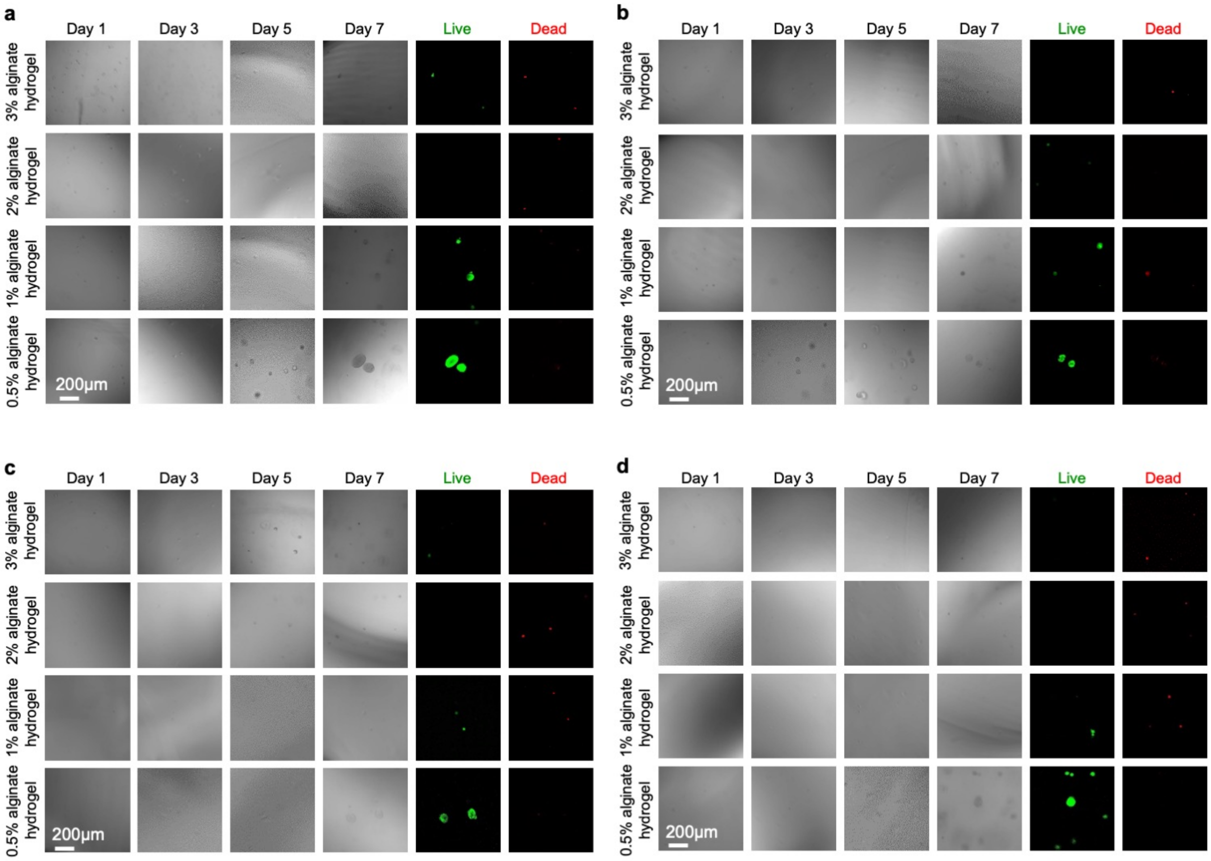

Figure S3. High concentrations of alginate hydrogel scaffolds inhibit the survival and proliferation of four different types of cancer cells. Bright field images of MDA-MB-231 (**a**), PC-3 (**b**), MCF-7 (**c**), and OVCAR-8 (**d**) cancer cells cultured in alginate hydrogel with 0.5, 1, 2, and 3% alginate in weight on days 1, 3, 5, and 7. Live/dead (green/red) staining shows negligible aggregate formation in the 2 and 3% alginate hydrogel scaffolds.

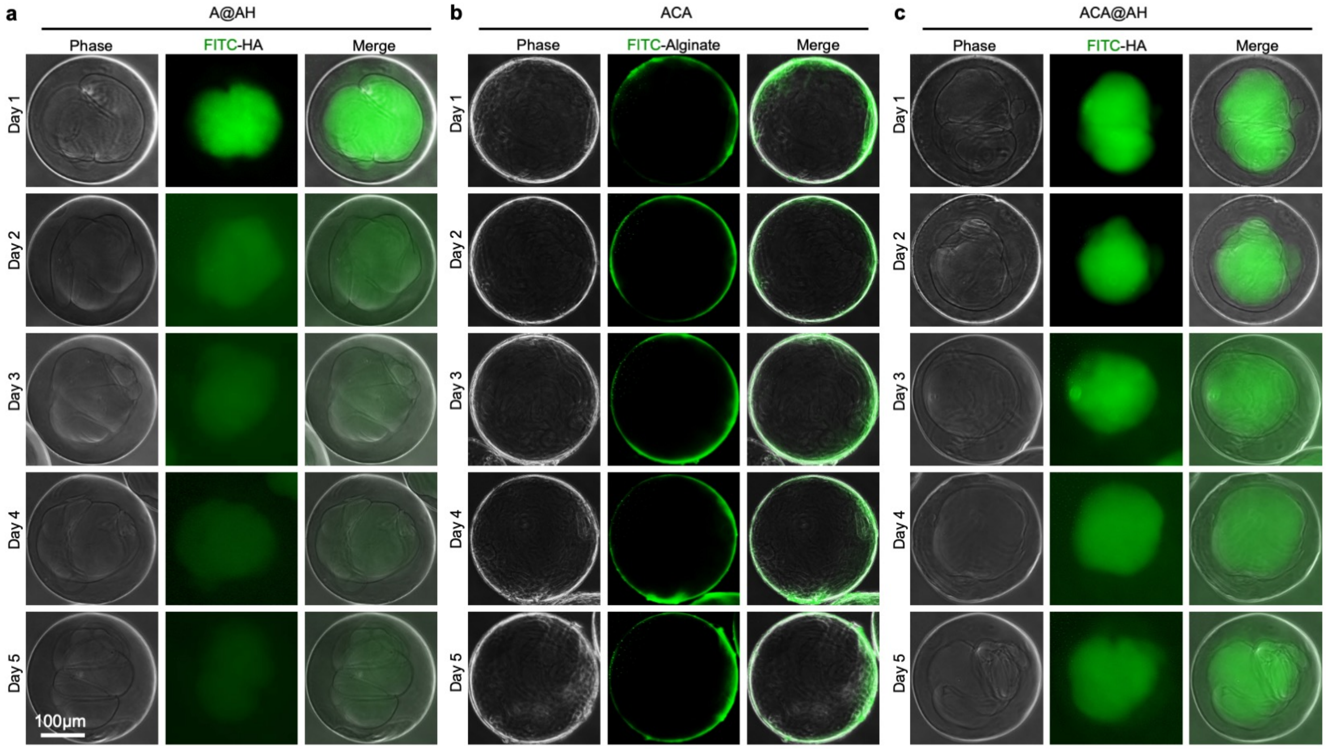

Figure S4. ACA coating retains HA inside the core of the core-shell microcapsules. **a**, FITC-labeled HA is used for the formation of microcapsules, showing that the HA is mainly distributed in the core immediately after microencapsulation. However, most of the HA gradually diffuses out of the microcapsules in 2 days. **b**, FITC-labeled alginate was used for the last coating step after the chitosan-coating step to make the ACA coating, showing successful formation of the ACA coating on the surface of the microcapsules. To focus on imaging the ACA coating, the core-shell structure of the microcapsules is not as evident in these images. **c**, Fluorescence images showing the ACA coating can help to retain HA inside the core of the microcapsules for at least for 5 days.

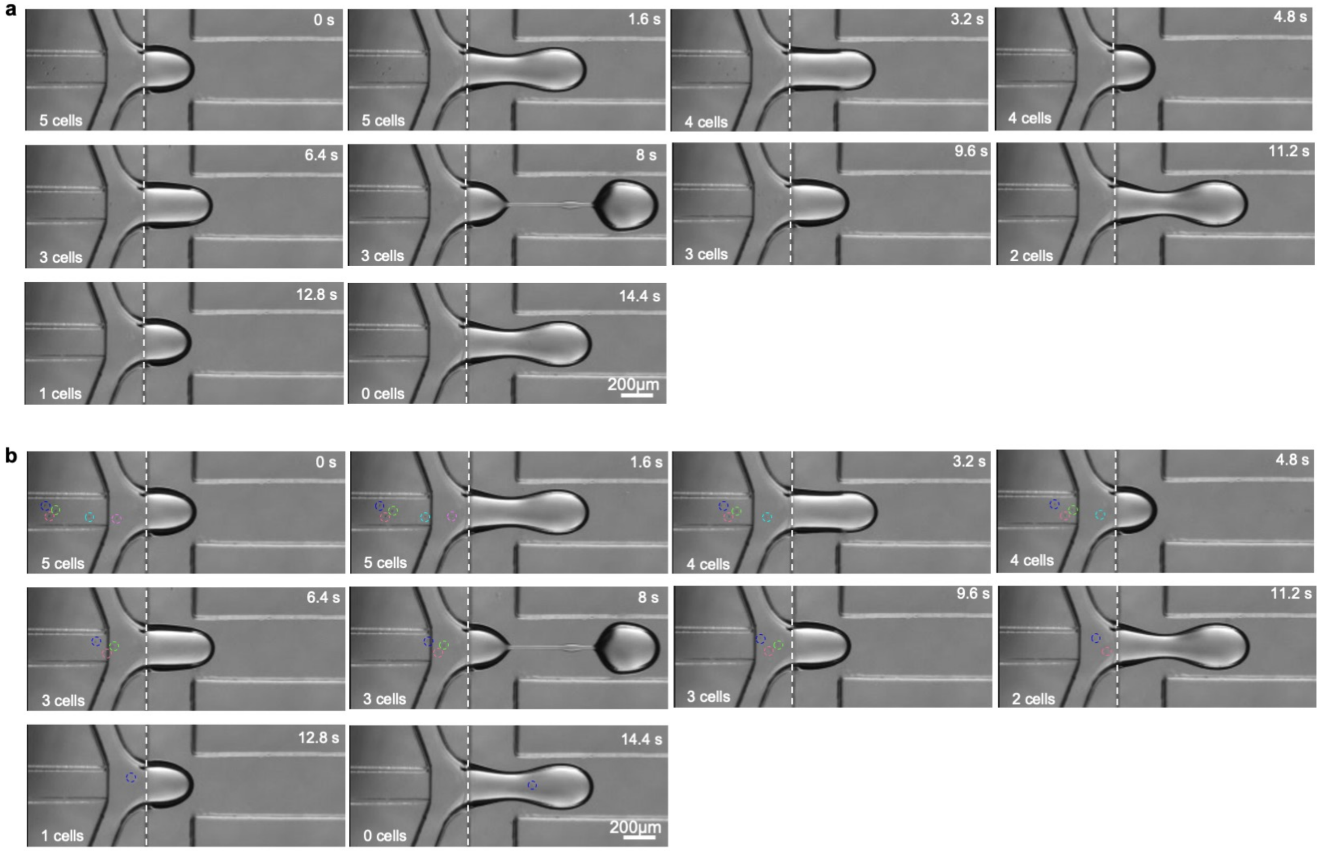

Figure S5. Time-sequenced bright-field micrographs showing the microfluidic process of encapsulating one single cell in each core-shell microcapsule. **a**-**b**, Micrographs either without (**a**) or with (**b**) dashed color circles to indicate the location of cells in the flow-focusing junction. In **b**, circles of the same color refer to the same cell in different images.

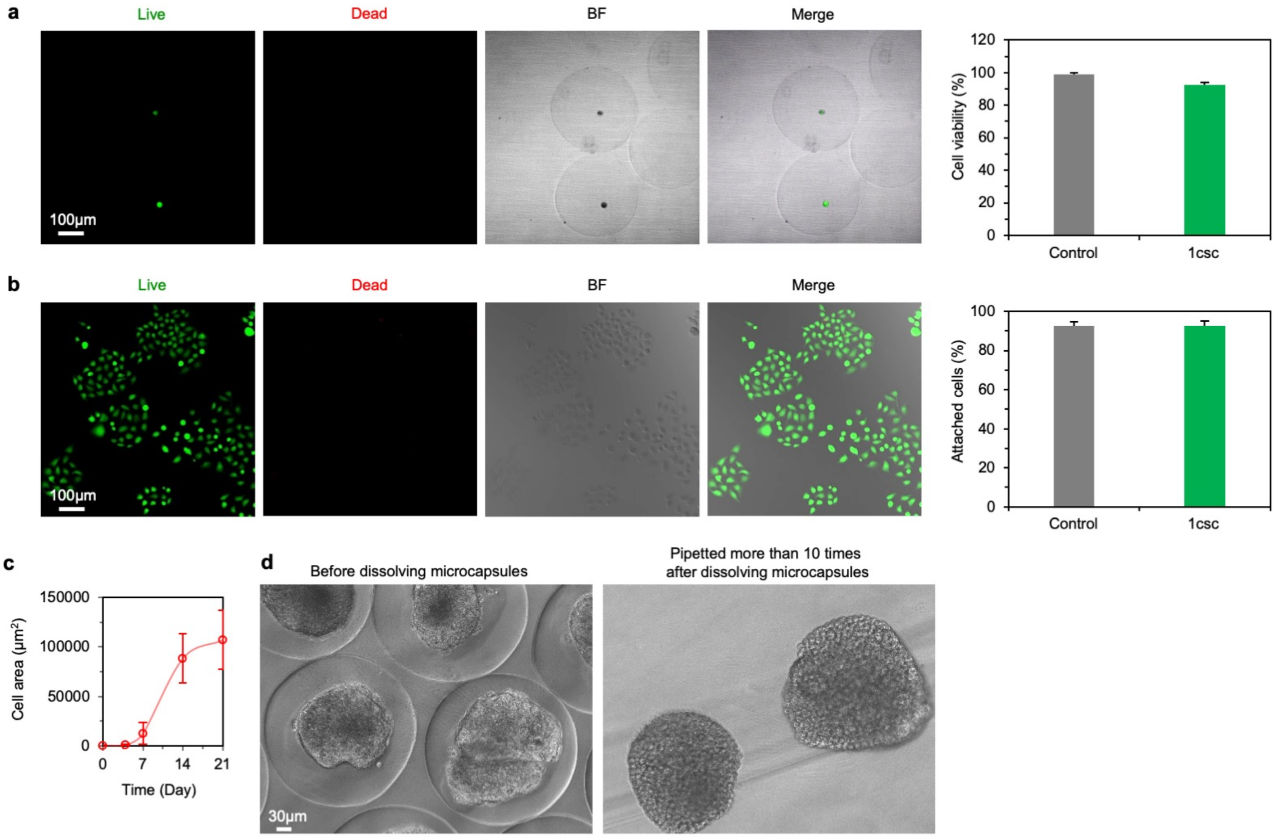

Figure S6. The one single cell in microcapsules is highly viable after the microencapsulation procedure and the colonies formed are highly stable. **a**, Confocal images and quantitative data show more than 90% of the cells are alive after microencapsulation. To focus on the cells, the core-shell structure of the microcapsules is not evident in these images. **b**, More than 90% of the cells could attach and proliferate after they were released out of the microcapsules using an isotonic solution of sodium citrate and pipetting. **c**, Proliferation of the encapsulated one single cell based on the area occupied by the cell/colony in the microcapsules determined by using the ImageJ software. **d**, Differential interference contrast (DIC) images show that the cell colonies are stable even after removing the microcapsule and pipetting for more than 10 times.

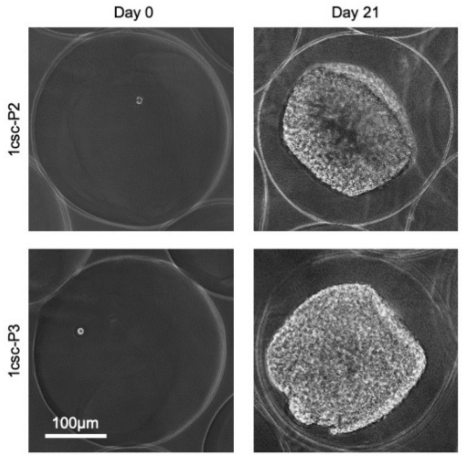

Figure S7. Representative phase contrast micrographs of second- and third-passage (1csc-P2 and 1csc-P3) colonies formed under the 1csc culture from one single cell. The single cells for making the 1csc-P2 and 1csc-P3 colonies were obtained by dissociating the first-passage 1csc (1csc-P1 that is the 1csc group in **Fig. 1e-f**) and second-passage (1csc-P2) colonies, respectively.

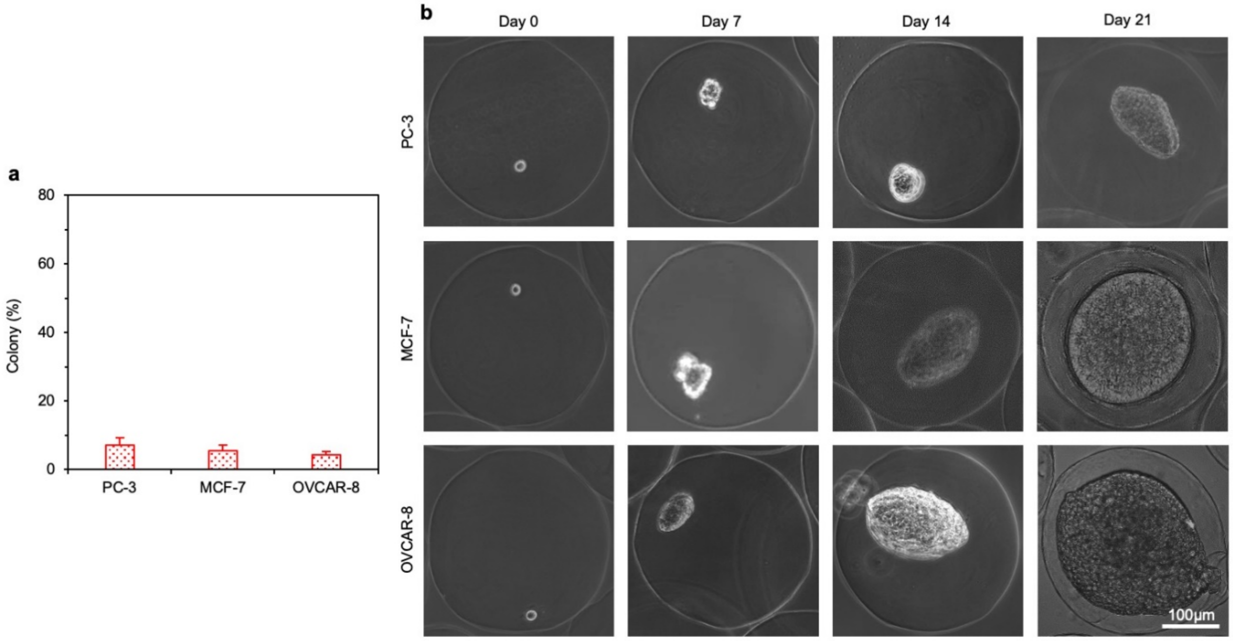

Figure S8. The bioinspired one single cell (1csc) culture method for CSC isolation and culture is applicable to different types of cancer cells. **a**, Percentage of colony formation of PC-3 human prostate cancer cells, MCF-7 human breast cancer cells ($HER2^{+}$), and OVCAR-8 human ovarian cancer cells formed after the 1csc culture. **b**, Representative micrographs showing the colony formation from one single cell in the microcapsule core and the growth of the colonies in three weeks for the three different types of cancer cells.

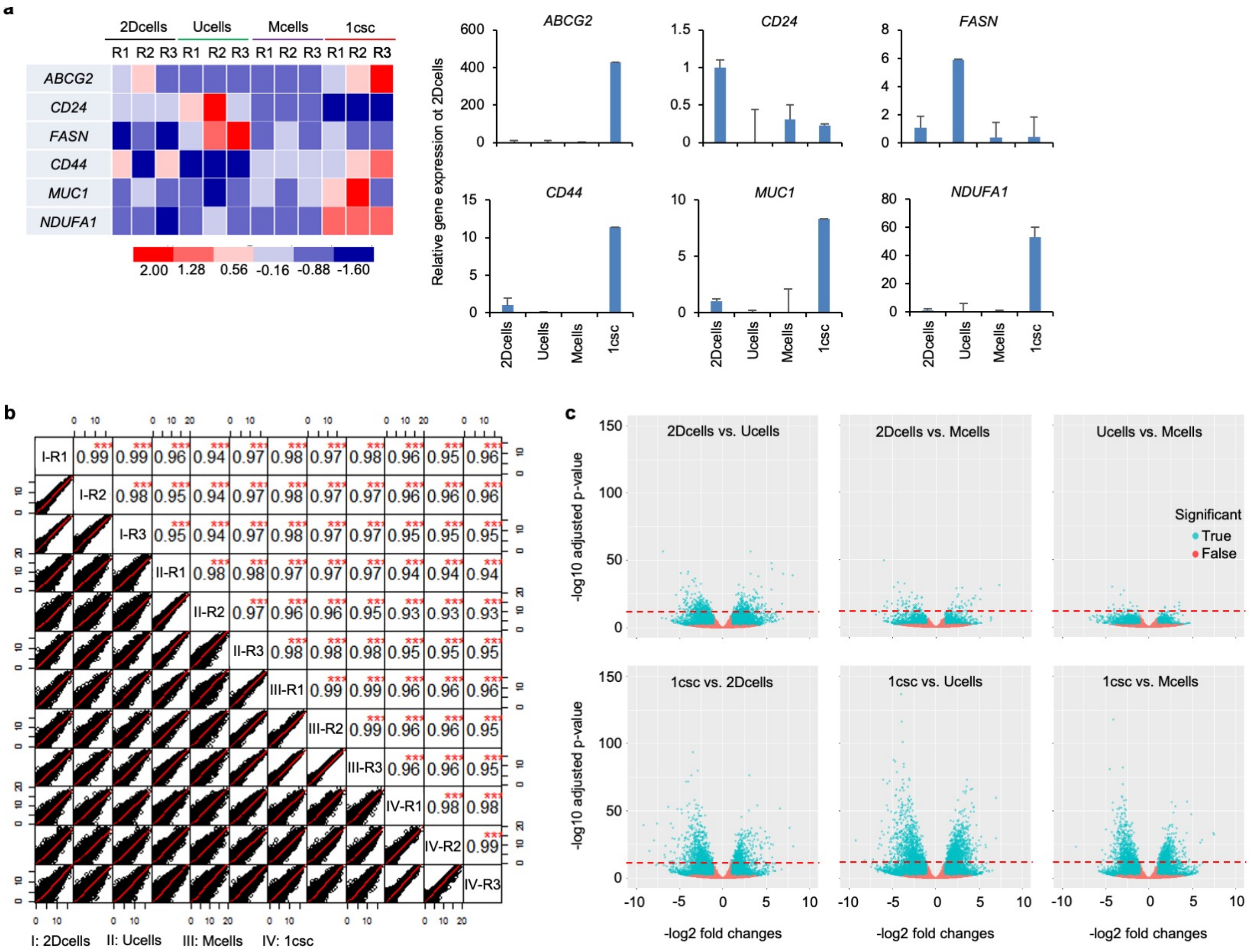

Figure S9. Validation and quality of the RNA sequencing data. **a**, Validation of RNA sequencing data (heat map, left) using RT-PCR (right) for six different genes. **b**, Pearson correlation matrix showing that all the triplicates and four culture methods are highly correlated (the diagonal of the heat map, correlation value: 0.97-0.99). **c**, Volcano plots showing the distribution of the differentially expressed genes of various pairs.

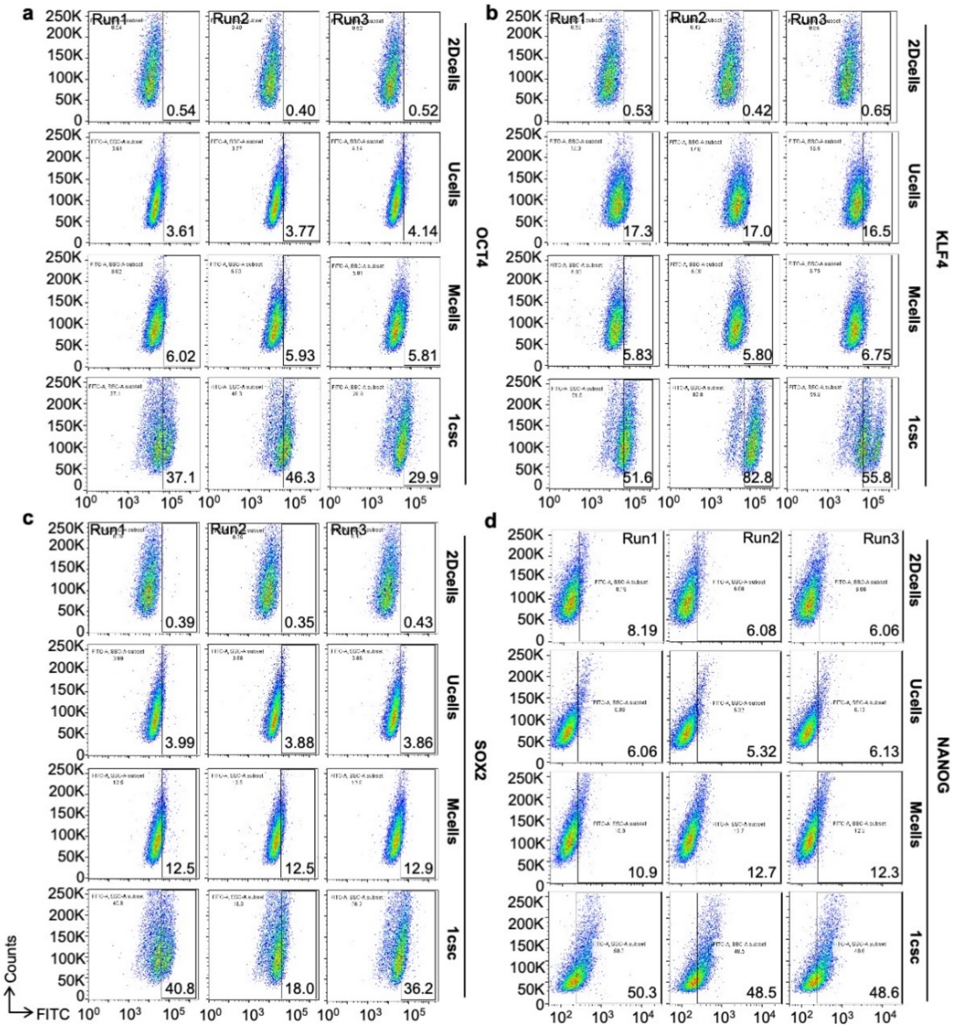

Figure S10. Stemness protein markers are highly expressed in the 1csc colony cells compared to cells in the 2Dcells, Ucells, and Mcells groups. **a**-**d**, Expression of OCT4 (**a**), KLF4 (**b**), SOX2 (**c**), and NANOG (**d**) proteins was analyzed in triplicates using flow cytometry, which shows that the 1csc colony cells have the highest expression of the four stemness protein markers among all the four groups.

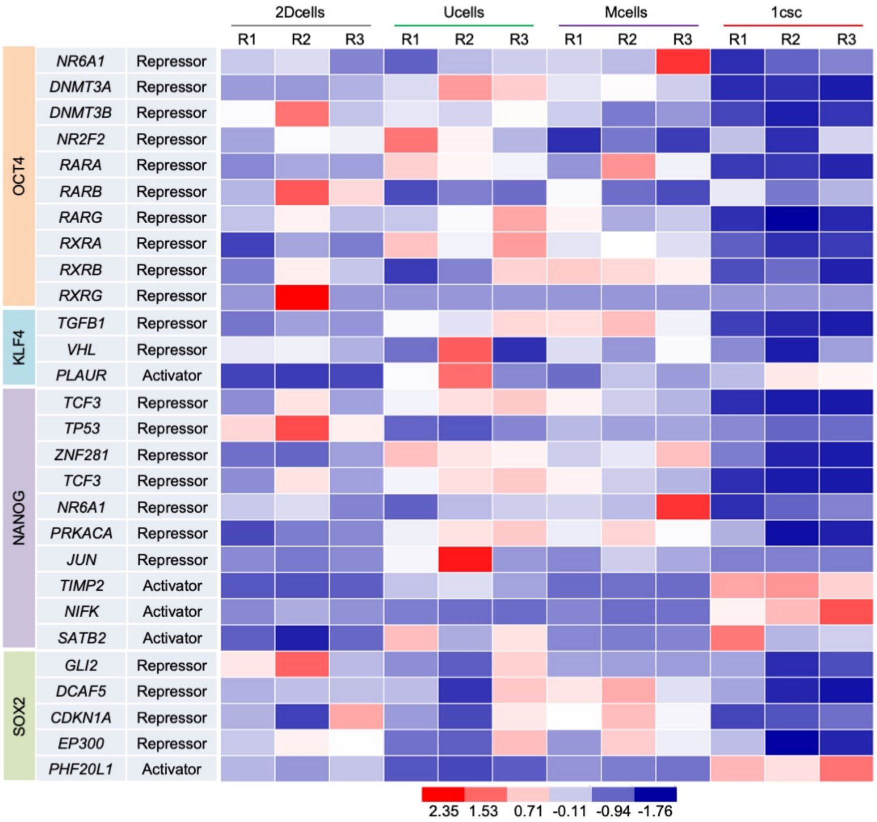

Figure S11. Heat map of repressor and activator genes for OCT4, KLF4, NANOG, and SOX2, showing that the repressor genes are downregulated whereas the activator genes are upregulated in the 1csc colony cells, which may contribute to the high expression of these protein markers in the 1csc colony cells as shown in **Fig. 2e-f** during the translation step of protein expression.

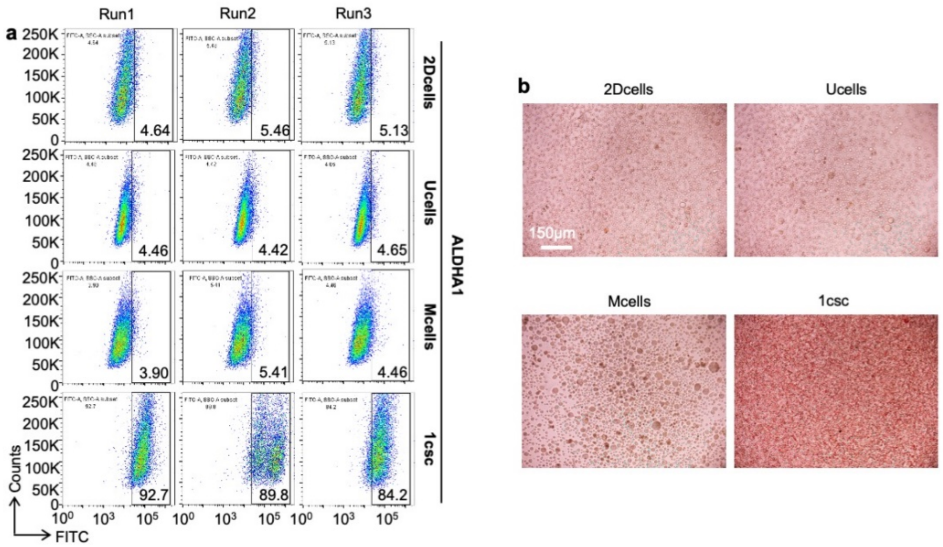

Figure S12. ALDHA1 or alkaline phosphatase (AP) are highly expressed in the 1csc colony cells compared to cells in the 2Dcells, Ucells, and Mcells groups. **a**, Expression of ALDHA1 was analyzed in triplicates using flow cytometry, which shows that the 1csc colony cells have the highest expression among all the four groups. **b**, Bright field micrographs of cells from the 2Dcells, Ucells, Mcells, and 1csc groups stained with alkaline phosphatase (AP) show its highest expression in the 1csc group.

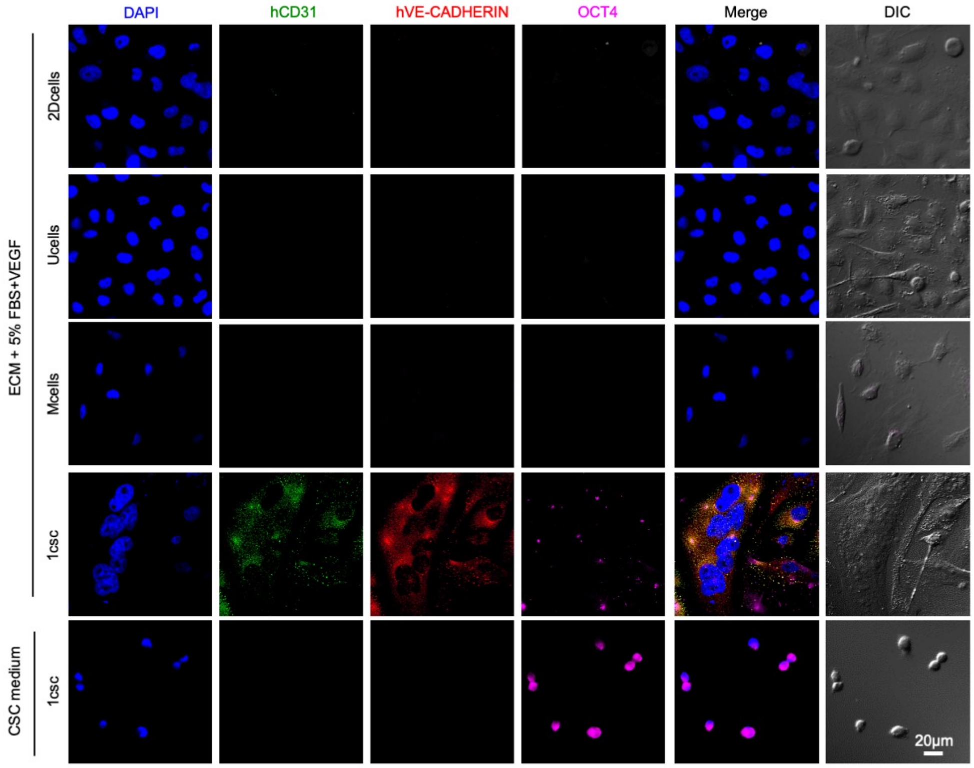

Figure S13. Confocal micrographs of endothelial differentiation showing the expression of human CD31 (hCD31) and human VE-CADHERIN (hVE-CADHERIN) in the cells differentiated from the 1csc colony cells. At the same time, the expression of OCT4 in the differentiated 1csc colony cells is downregulated. In contrast, the 1csc colony cells cultured in CSC medium do not express the two endothelial markers and has high expression of OCT4. All the three protein markers are negligible in the differentiated cells of the 2Dcells, Ucells, and Mcells groups.

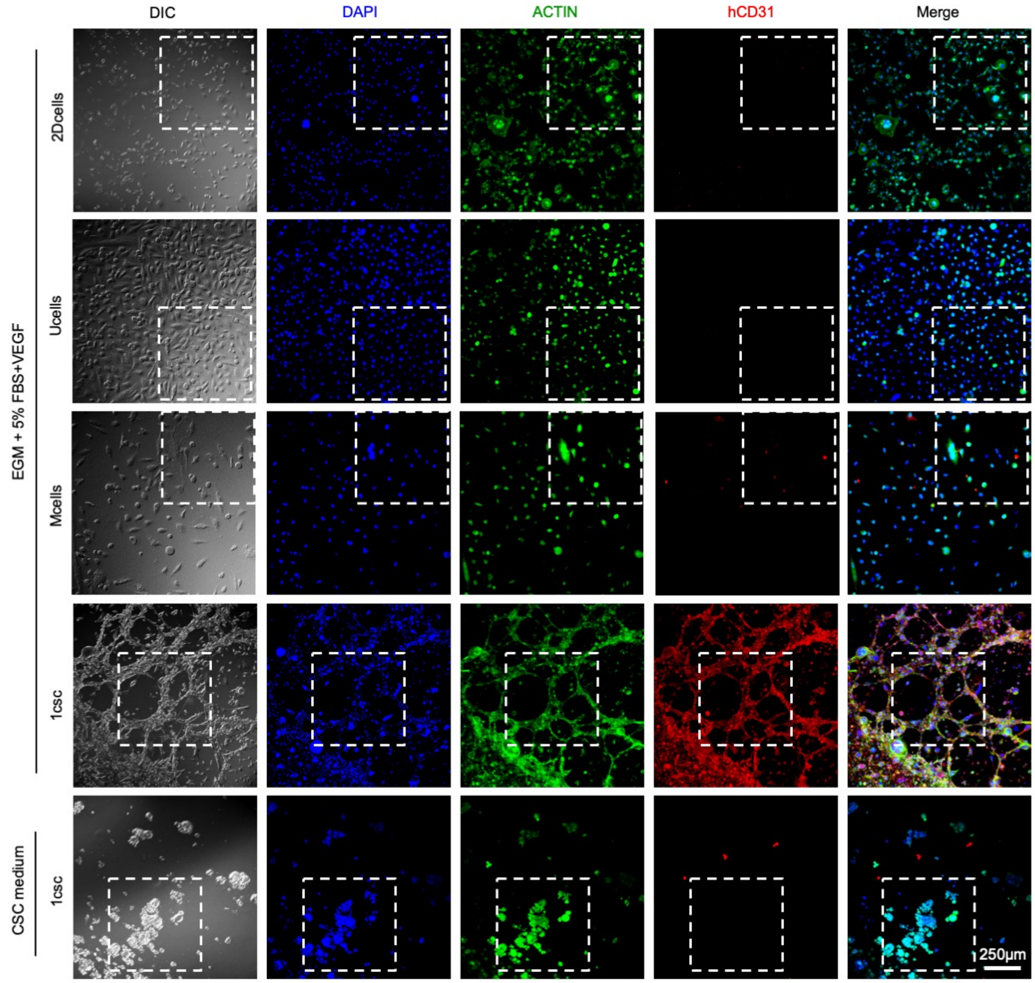

Figure S14. DIC and fluorescence images of ACTIN and hCD31 showing successful blood vessel-like tube formation when culturing the 1csc colony cells on Matrigel with endothelial growth medium (EGM) supplemented with 5% FBS and 50 ng ml^{-1} VEGF. In contrast, the 1csc colony cells form aggregates when they are cultured in the CSC medium. The boxed areas are shown in **Fig. 3b**.

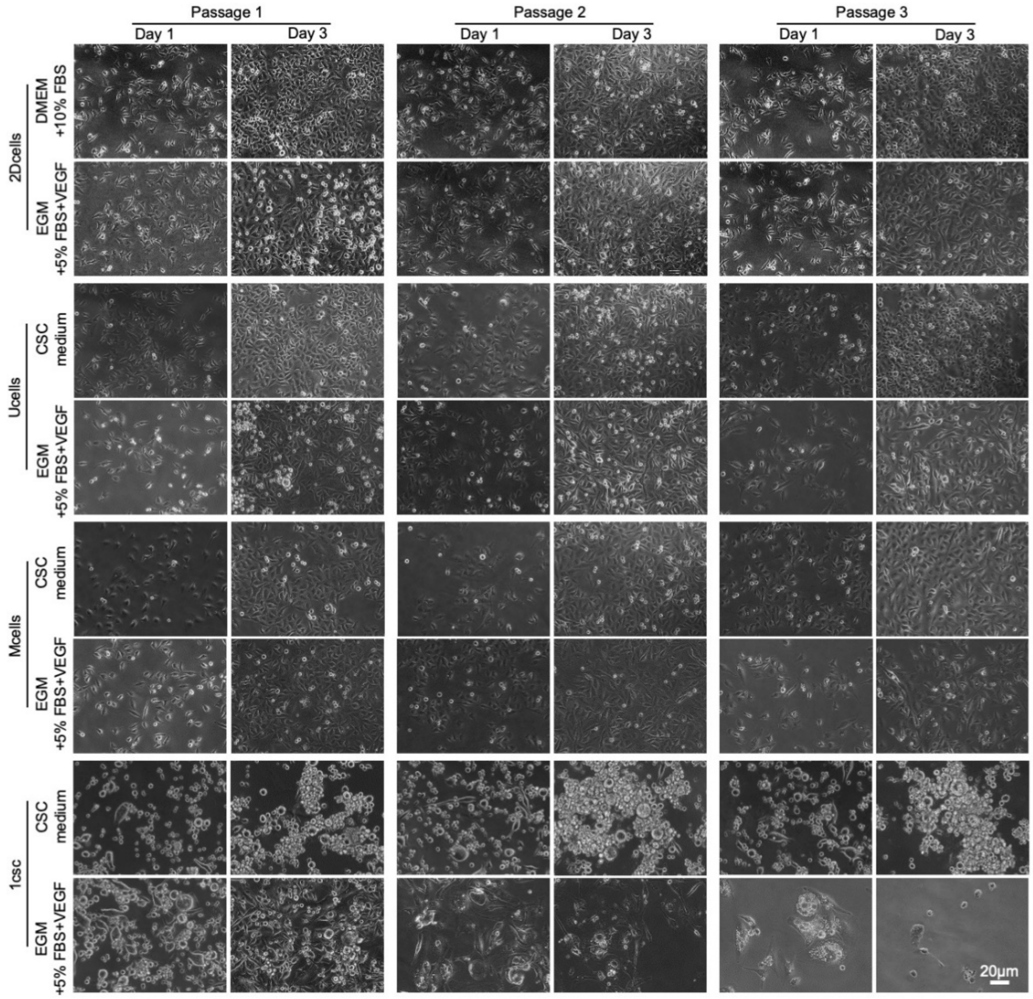

Figure S15. Phase contrast micrographs of cells from the 2Dcells, Ucells, Mcells, and 1csc groups during culturing in CSC medium, endothelial growth medium (EGM) supplemented with 5% FBS and 50 ng ml^{-1} VEGF, or DMEM supplemented with 10% FBS (for 2Dcells only) for up to 3 passages, showing cells from the 2Dcells, Ucells, and Mcells groups proliferate through passage 3. In contrast, most of the cells from the 1csc group cannot survive after the second passage although they survive well in the CSC medium. It is also observed that cells from the 1csc group tend to form aggregates when cultured in the CSC medium.

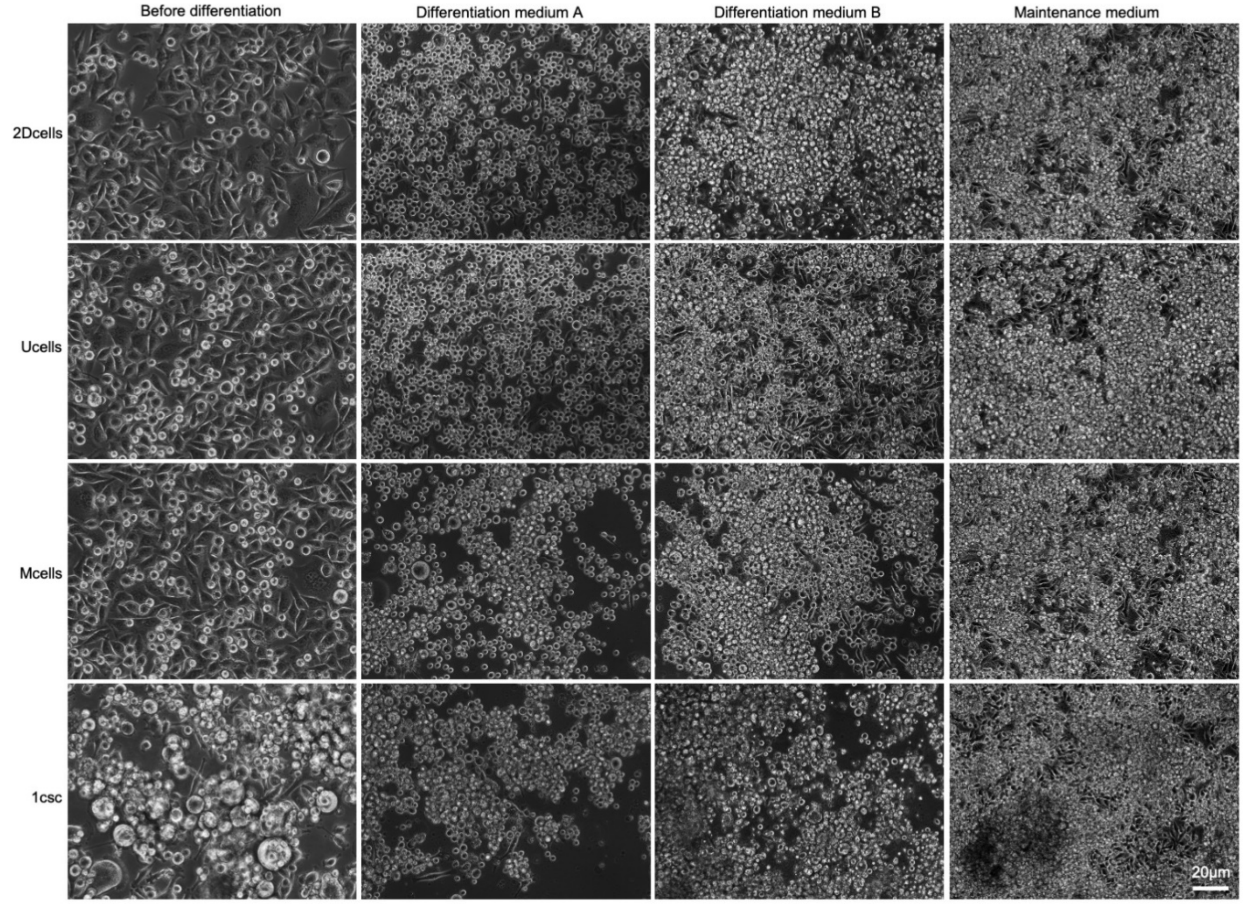

Figure S16. Phase contrast micrographs showing the morphology of cells from the 2Dcells, Ucells, Mcells, and 1csc groups during cardiac differentiation.

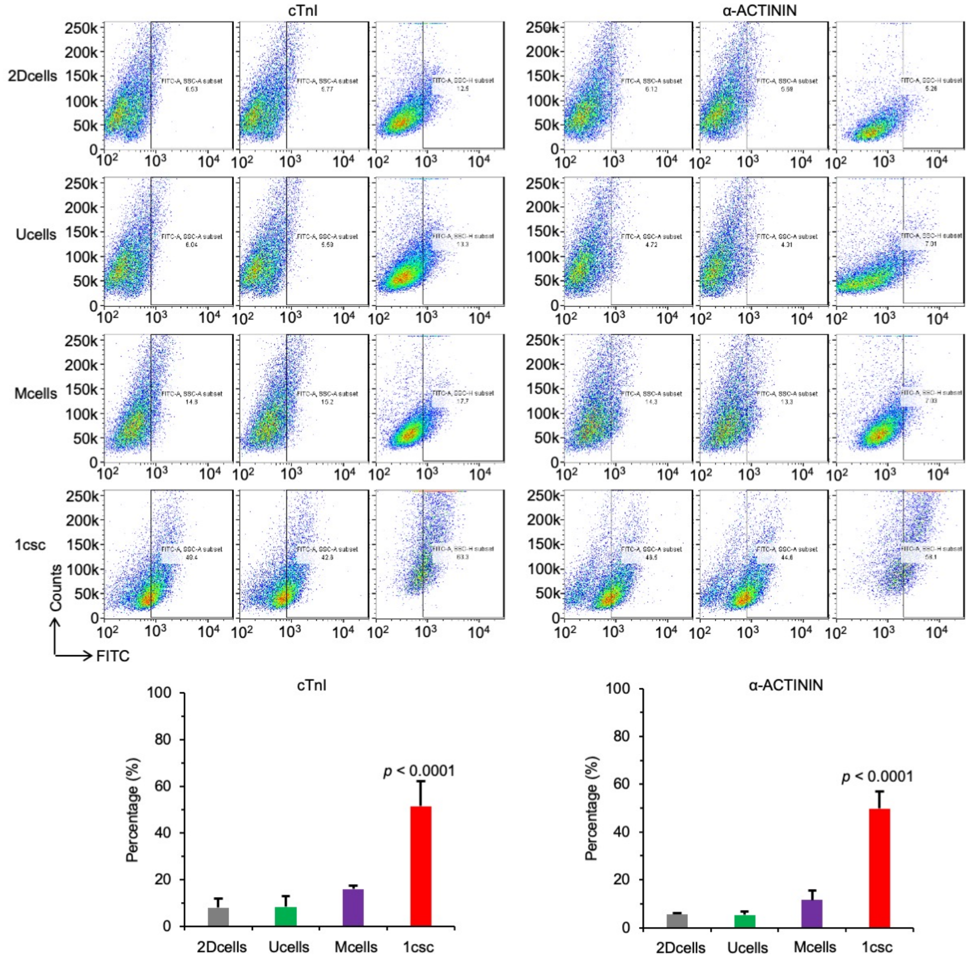

Figure S17. Cardiac specific markers (cTnI and α-ACTININ) are highly expressed in cells after cardiac differentiation in the 1csc group in comparison to the 2Dcells, Ucells, and Mcells groups. Flow cytometry of three independent runs and quantitative results show significantly higher expression of the cardiac specific markers in the 1csc group than the 2Dcells, Ucells, and Mcells groups. Error bars denote mean ± s.d., and statistical significance was assessed by one-way ANOVA with post hoc Tukey test. .

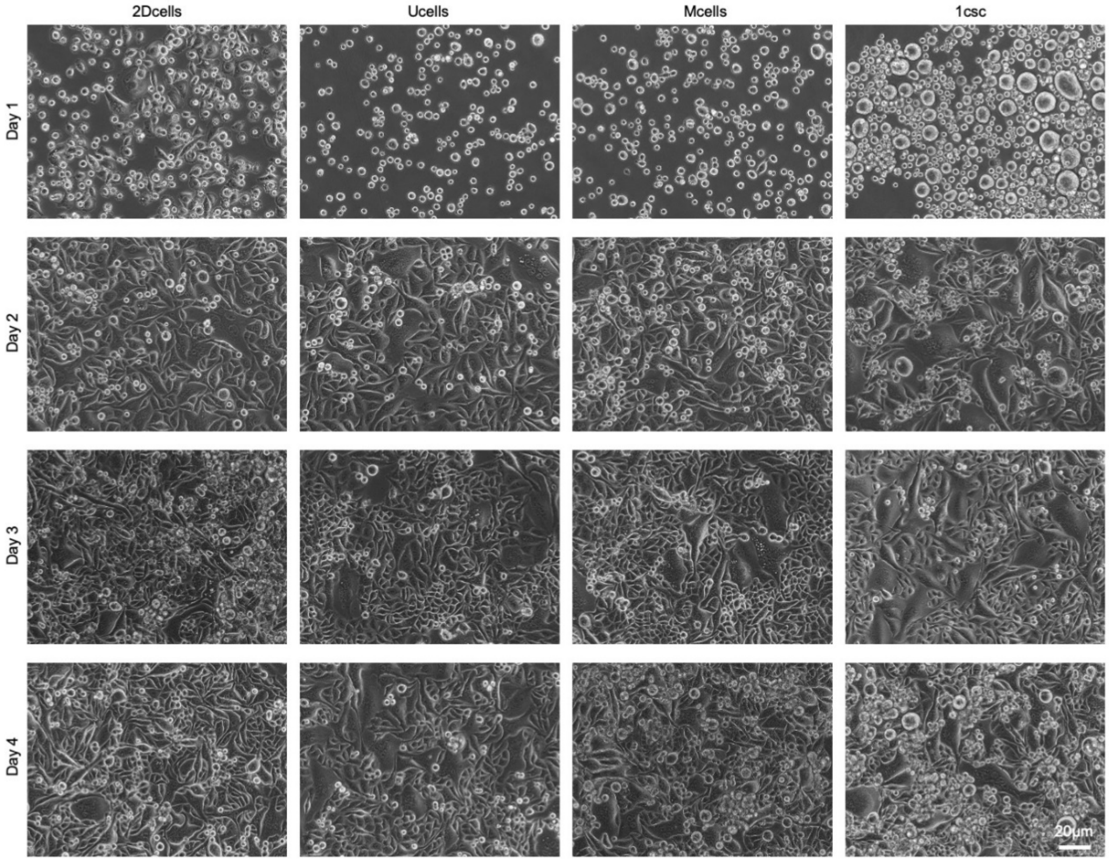

Figure S18. Phase contrast micrographs showing the morphology of cells from 2Dcells, Ucells, Mcells, and 1csc groups during osteogenic differentiation.

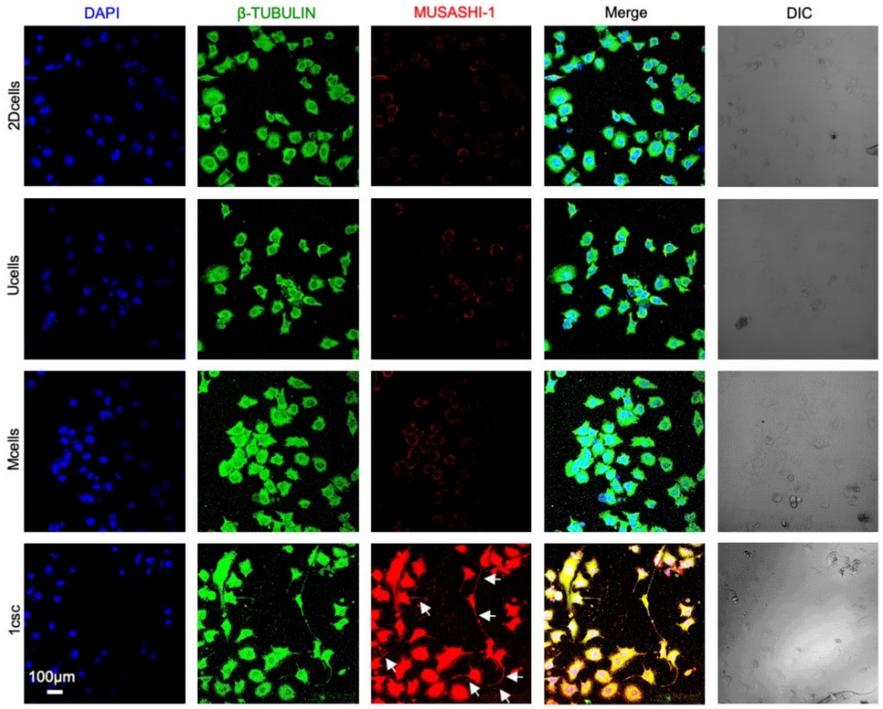

Figure S19. Confocal micrographs of the cells from the 2Dcells, Ucells, Mcells, and 1csc groups after neural differentiation show significantly higher expression of the neural specific marker MUSASHI-1 in the 1csc group than the other three groups. The β-TUBULIMN was stained to show the cytoskeleton of the cells. White arrows indicate neurites of the differentiated cells, which could be seen only in the 1csc group.

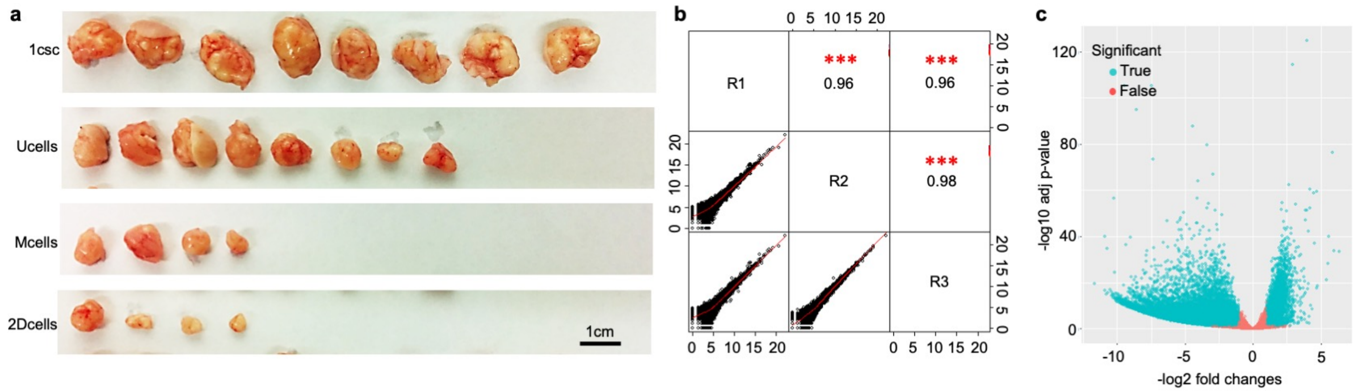

Figure S20. Photographs of tumors obtained on day 55 and RNA-Seq analyses of cells isolated from *in vivo* tumors. **a**, Photographs showing significantly larger tumors in the 1csc group than the other three groups. Tumor formation occurs in all the eight mice of the 1csc and Ucells groups, but it occurs in only 4 of the eight mice for both the 2Dcells and Mcells groups. **b**, Pearson correlation matrix showing high correlation in biological replicates (R1, R2, and R3) of the gene sequencing data on cells in tumors of the 1csc group (the diagonal of the heat map, correlation value: 0.96-0.98). **c**, Volcano plot showing differentially expressed genes between the *in vitro* 1csc colony cells (before injection into mice) and cells in *in vivo* tumors grown from the 1csc colony cells injected into the fat pads of mice.

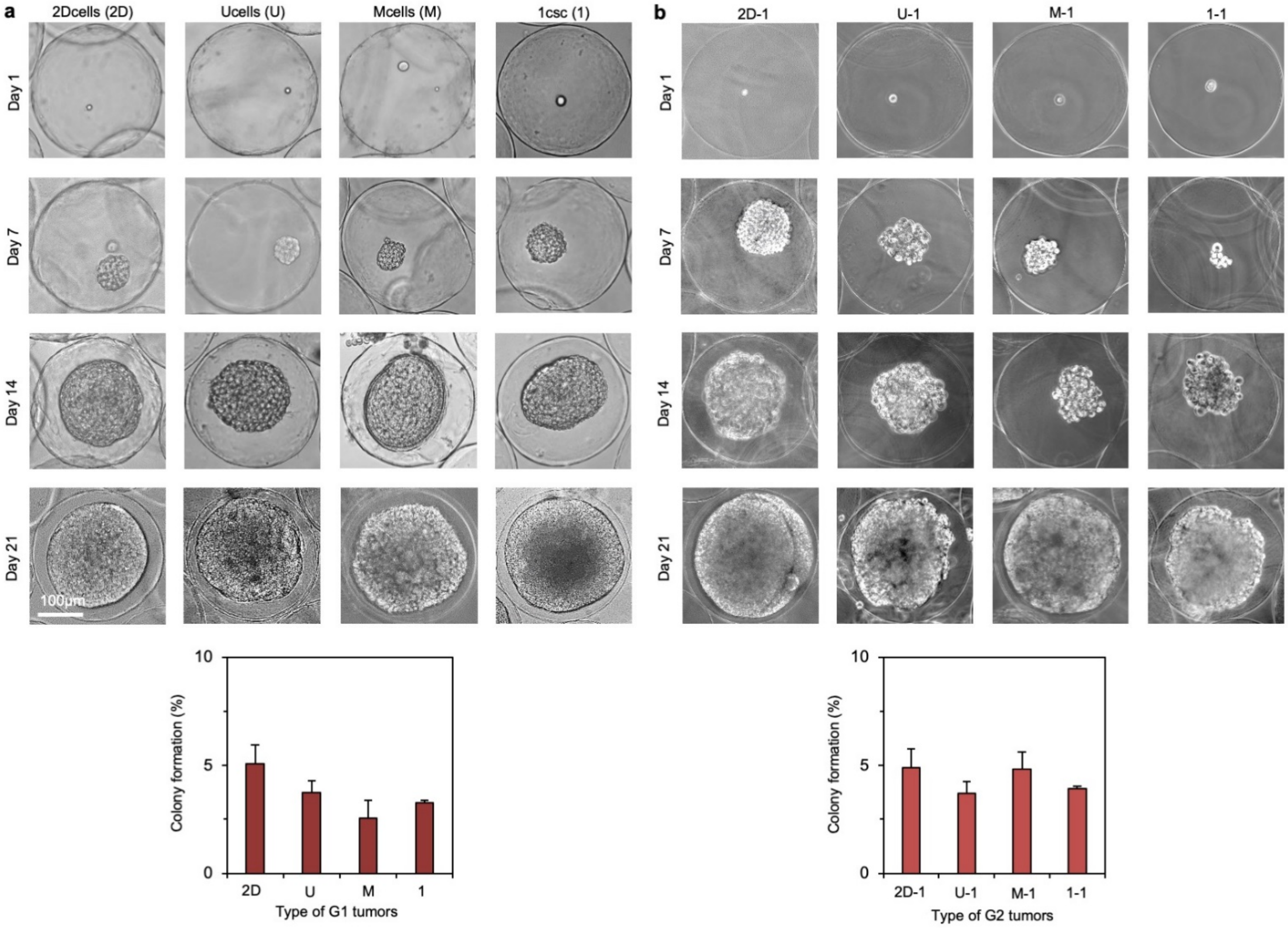

Figure S21. One single cell microencapsulation of cells isolated from *in vivo* tumors for 1csc culture. **a**, Bright field micrographs showing the proliferation of the one single cell isolated from G1 tumors of the 2Dcells, Ucells, Mcells, and 1csc groups under the 1csc culture. Approximately 4% of the tumor cells could form colonies, which is similar to the parent MDA-MB-231 cell line, suggesting that the percentage of CSCs in the *in vivo* tumors is similar to that in the 2D cultured parent cells. **b**, Bright field micrographs showing the proliferation of one single cell isolated from G2 tumors of the 2D-1, U-1, M-1, and 1-1 groups under the 1csc culture. Similarly, the percentage of colony formation is ~4% for all the four groups, which is consistent with the results obtained with the G1 tumors. Error bars denote mean ± s.d.

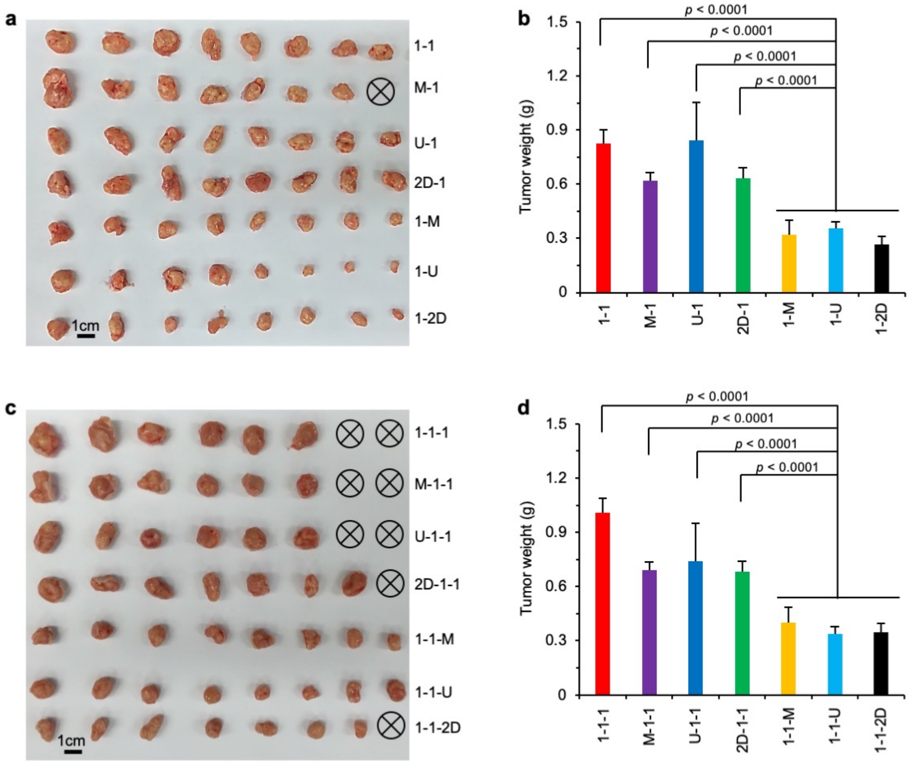

Figure S22. *In vivo* tumorigenesis of cells obtained by 2Dcells, Ucells, Mcells, and 1csc culture of cells isolated from *in vivo* tumors. **a-b**, Tumor image (**a**) and weight (**b**) showing that the 1-1, M-1, U-1 and 2D-1 G2 tumors are significantly larger than the 1-U, 1-M, and 1-2D tumors on day 55. This suggests the 1csc colony cells are more tumorigenic than the cells obtained with the 2Dcells, Ucells, and Mcells cultures regardless of the types of G1 tumors. **c-d**, Tumor image (**c**) and weight (**d**) of G3 tumors showing the cells isolated from the G2 *in vivo* tumors with the 1csc culture could form larger tumors than the cells isolated from G2 *in vivo* tumors with the 2Dcells, Ucells, and Mcells cultures. Error bars denote mean ± s.d., and n = 8 for each group except for M-1 group (n = 7), 1-1-1 (n = 6), M-1-1 (n = 6), U-1-1 (n = 6), 2D-1-1 (n = 7), and 1-1-2D (n = 7) due to animal death. Statistical analyses were performed by one-way ANOVA with post hoc Tukey test. The 1-1, M-1, U-1, or 2D-1 group is compared with the 1-M, 1-U, and 1-2D groups altogether. The 1-1-1, M-1-1, U-1-1, or 2D-1-1 group is compared with the 1-1-M, 1-1-U, and 1-1-2D groups altogether. The symbol ⊗ indicates death of animal (note: the survival of animal is not statistically significant between the various groups).

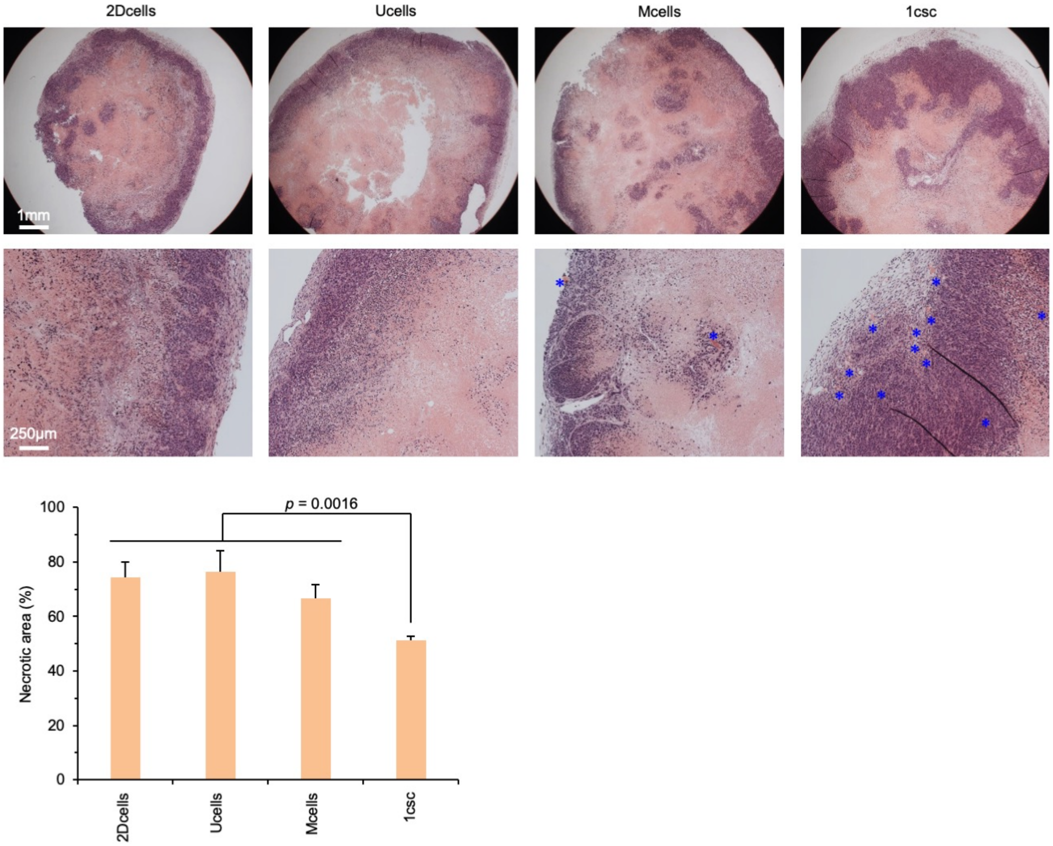

Figure S23. Significantly increased formation of blood vessels and decreased area of necrosis in G1 tumors of the 1csc group. Representative images of hematoxylin and eosin (H&E) staining of G1 tumor tissues collected after sacrificing mice on day 55 for the 2Dcells, Ucells, Mcells, and 1csc groups. Quantitative analyses show that tumors of the 1csc group have significantly less necrotic area than tumors in the other three groups. This is potentially due to the greater capability of the 1csc colony cells in differentiating into endothelial cells to form blood vessels indicated by blue asterisks. Error bars denote mean ± s.d., n = 3. Statistical analyses were performed by one-way ANOVA with post hoc Tukey test. The 1csc group is compared with the 2Dcells, Ucells, and Mcells groups altogether.

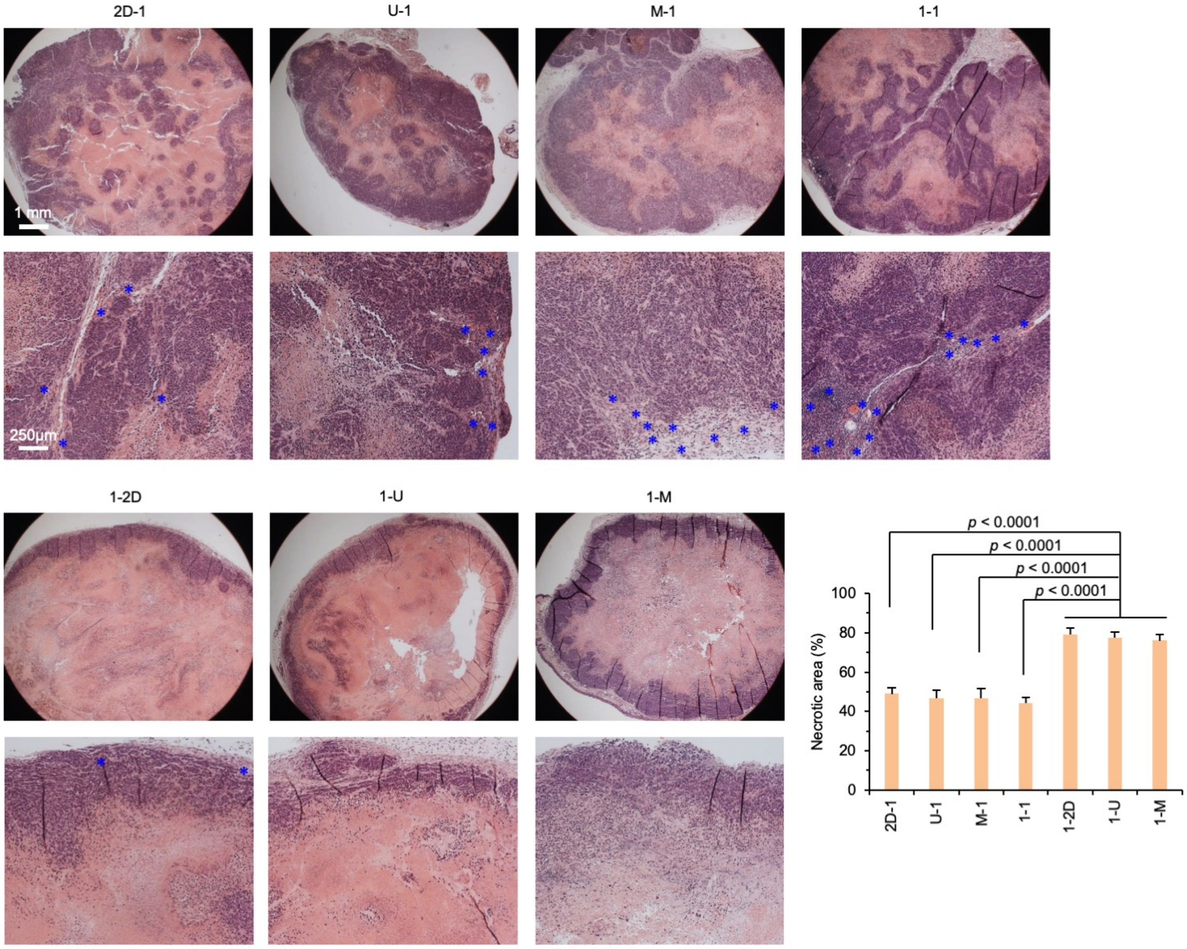

Figure S24. Significantly increased formation of blood vessels and decreased area of necrosis in G2 tumors grown from cells obtained using the 1csc culture method. Representative images of H&E stained G2 tumor tissues collected after sacrificing the mice on day 55 for the 2D-1, U-1, M-1, 1-1, 1-2D, 1-U, and 1-M groups. Quantitative analyses show that the G2 tumors grown from cells obtained with the 1csc culture (the 2D-1, U-1, M-1, and 1-1 groups) have less necrotic area than G2 tumors grown from cells obtained with 2Dcells (1-2D group), Ucells (1-U group), and Mcells (1-M group) cultures. Blue asterisks indicate blood vessels in tumor tissues, showing more blood vessels in the 2D-1, U-1, M-1, and 1-1 tumors than the 1-2D, 1-U, and 1-M tumors. Error bars denote mean ± s.d., n = 3, and statistical analyses were performed by one-way ANOVA with post hoc Tukey test. The 2D-1, U-1, M-1, or 1-1 group is compared with the 1-2D, 1-U, and 1-M groups altogether.

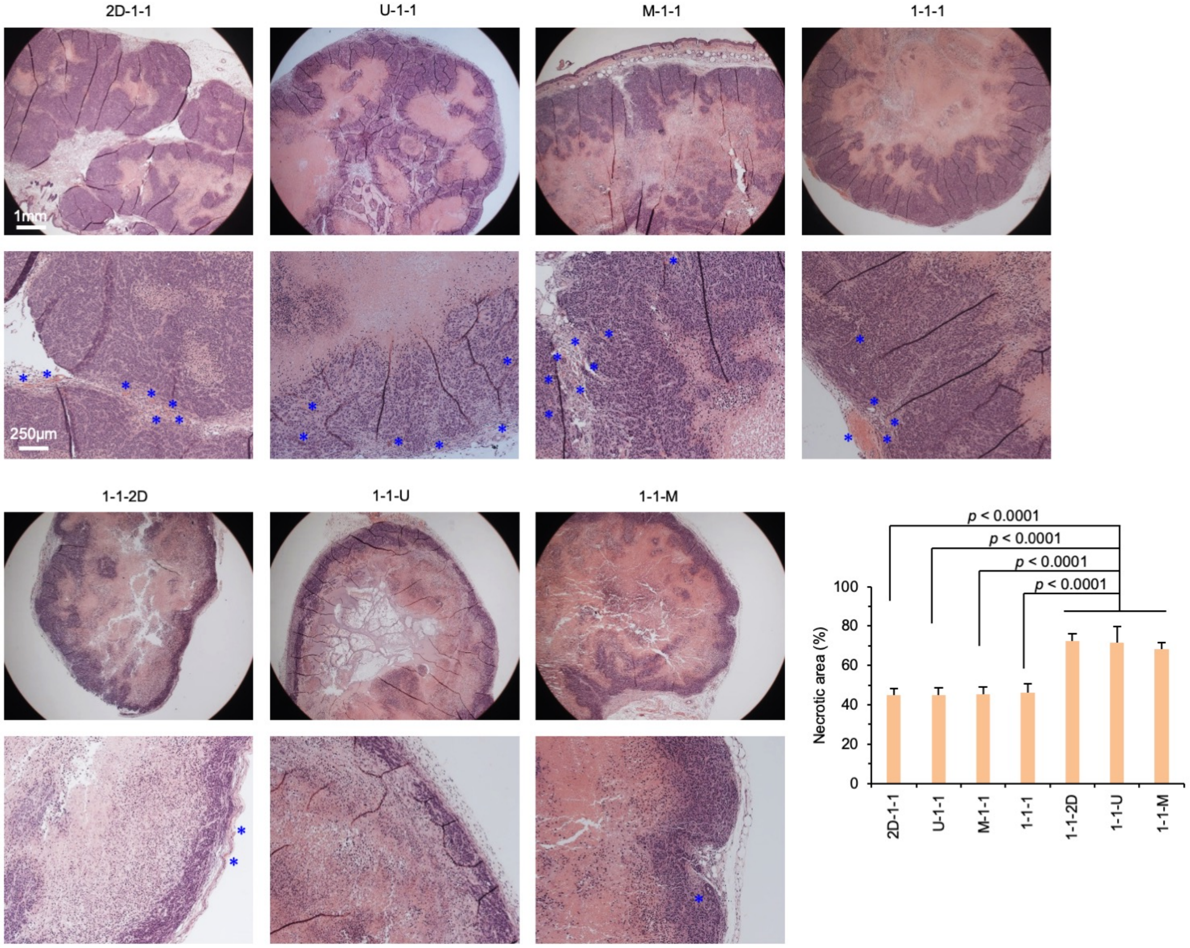

Figure S25. Significantly increased formation of blood vessels and decreased area of necrosis in G3 tumors grown from cells obtained using the 1csc culture method. Representative images of H&E stained G3 tumor tissues collected after sacrificing the mice on day 55 for the 2D-1-1, U-1-1, M-1-1, 1-1-1, 1-1-2D, 1-1-U, and 1-1-M. Quantitative analyses show that the G3 tumors grown from cells obtained with the 1csc culture (the 2D-1-1, U-1-1, M-1-1, and 1-1-1 groups) have significantly less necrotic area than G3 tumors grown from cells obtained with 2Dcells (1-1-2D group), Ucells (1-1-U group), and Mcells (1-1-M group) cultures. Blue asterisks indicate blood vessels in the tumor tissues, showing more blood vessels in the 2D-1-1, U-1-1, M-1-1, and 1-1-1tumors than the 1-1-2D, 1-1-U, and 1-1-M tumors. Error bars denote mean ± s.d., n = 3, and statistical analyses were performed by one-way ANOVA with post hoc Tukey test. The 2D-1-1, U-1-1, M-1-1 or 1-1-1 group is compared with the 1-1-2D, 1-1-U, and 1-1-M groups altogether.

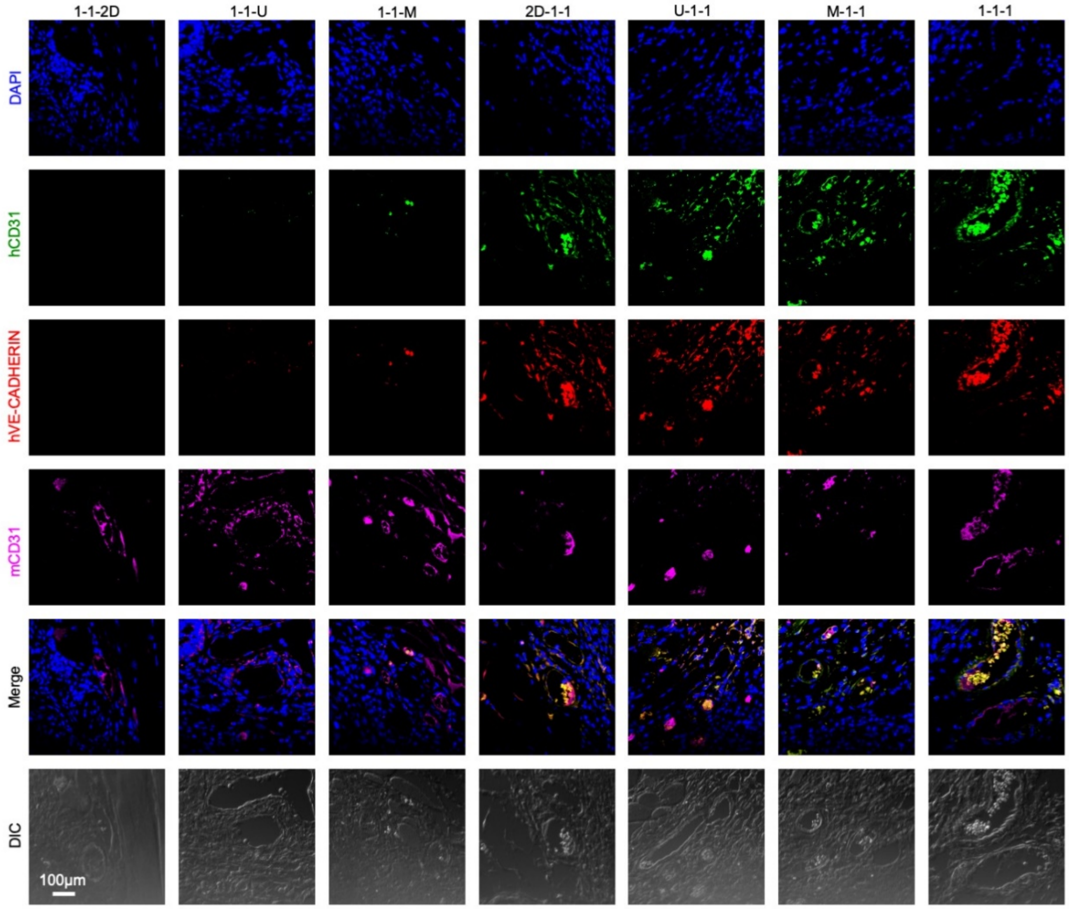

Figure S26. The 1csc colony cells differentiate into endothelial cells to form blood vessels in *vivo*. To identify the origin of the blood vessels in 1-1-2D, 1-1-U, 1-1-M, 2D-1-1, U-1-1, M-1-1, and 1-1-1 tumors, immunostainings with human CD31 (hCD31), human VE-CADHERIN (hVE-CADHERIN) and mouse CD31 (mCD31) were performed. The confocal micrographs show that blood vessels with hCD31and hVE-cadherin are evident in the 2D-1-1, U-1-1, M-1-1, and 1-1-1tumors grown from the 1csc colony cells. In contrast, staining of hCD31 or hVE-CADHERIN is negligible in 1-1-2D, 1-1-U, and 1-1-M tumors grown from cells obtained using the 2Dcells, Ucells, and Mcells culture methods, respectively. The mCD31 staining is observable in all the tumors.

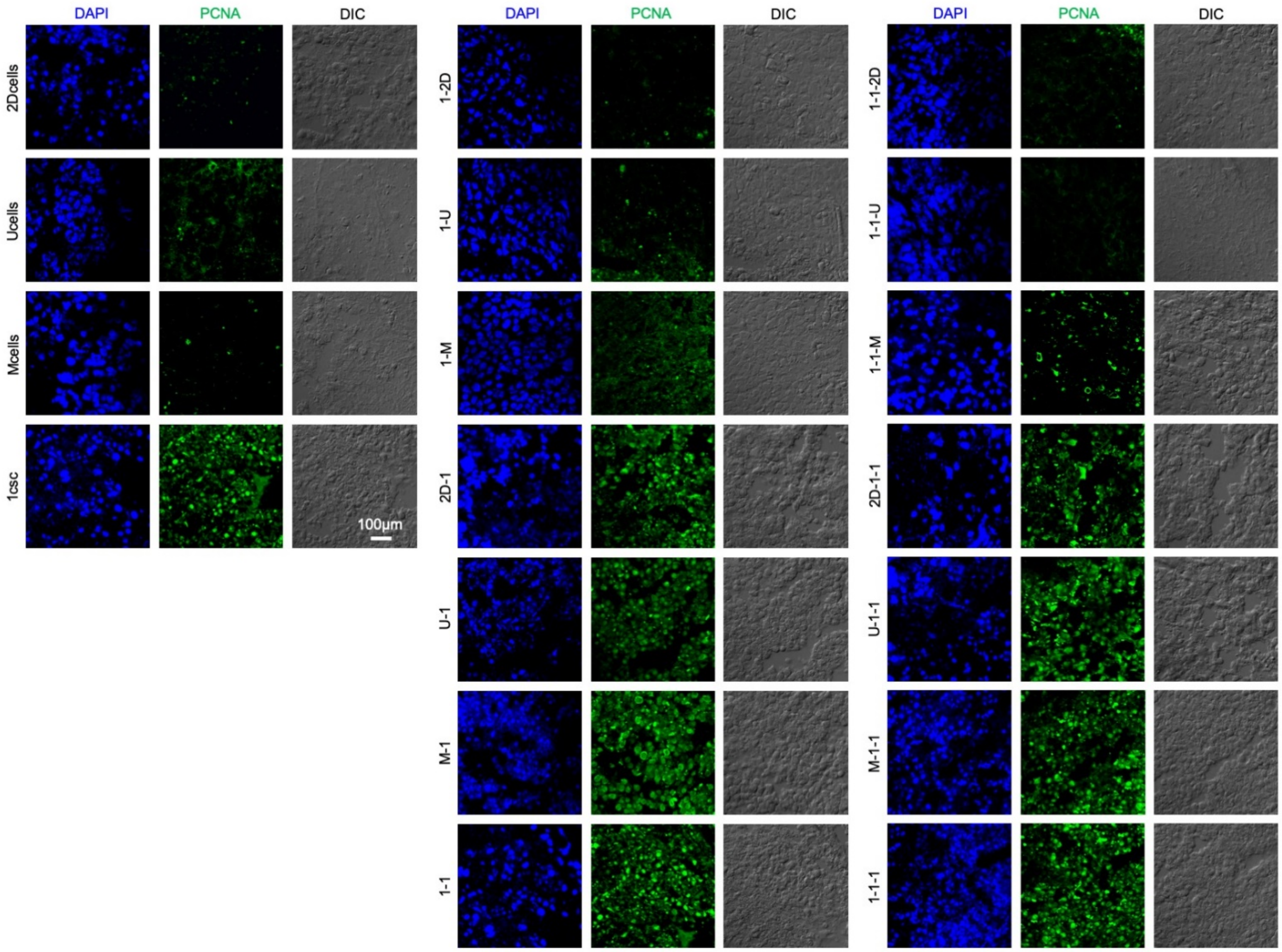

Figure S27. Multiple mechanisms may contribute to the fast growth of tumors from the 1csc colony cells. Confocal images showing that tumors (2D-1, U-1, M-1, 1-1, 2D-1-1, U-1-1, M-1-1, and 1-1-1) grown from the 1csc colony cells express more proliferating cell nuclear antigen (PCNA that promotes cell proliferation) than tumors (2Dcells, Ucells, Mcells, 1-2D, 1-U, 1-M, 1-1-2D, 1-1-U, and 1-1-M) grown from cells obtained with the other three culture methods (i.e., 2Dcells, Ucells, and Mcells).

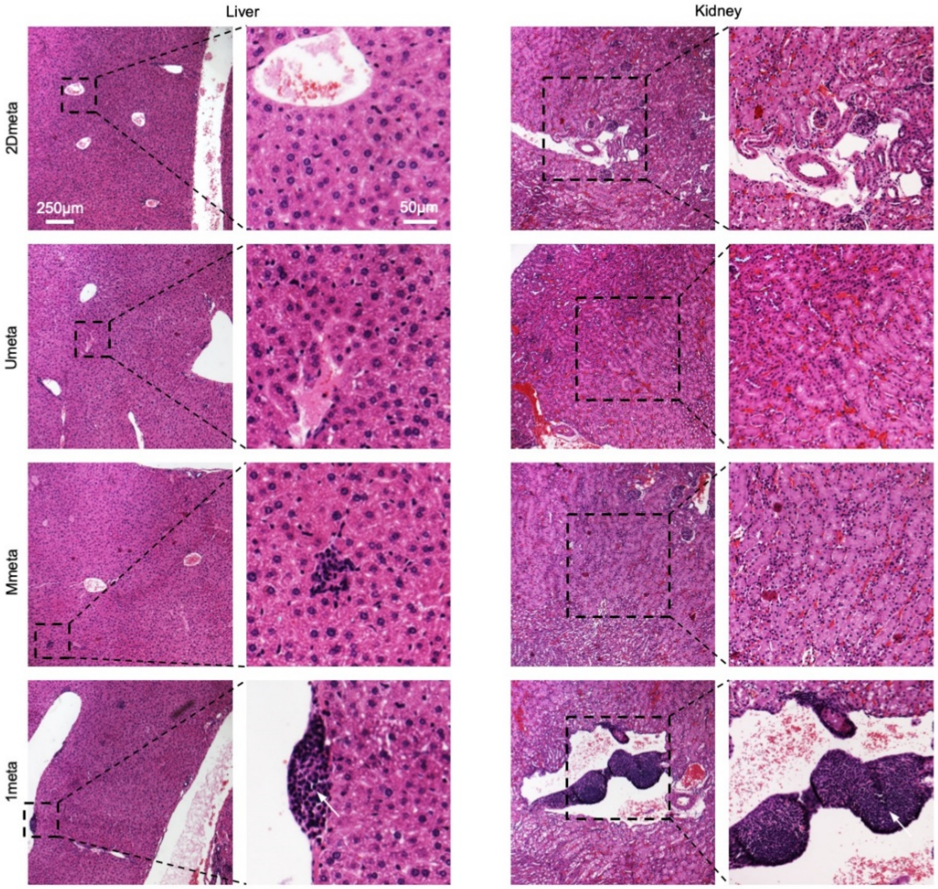

Figure S28. Histology (H&E staining) micrographs show that the 1csc colony cells may metastasize in livers and kidneys to form metastasis (1meta) in the two critical organs (arrows in the zoom-in images at the bottom row) after intravenous injection.

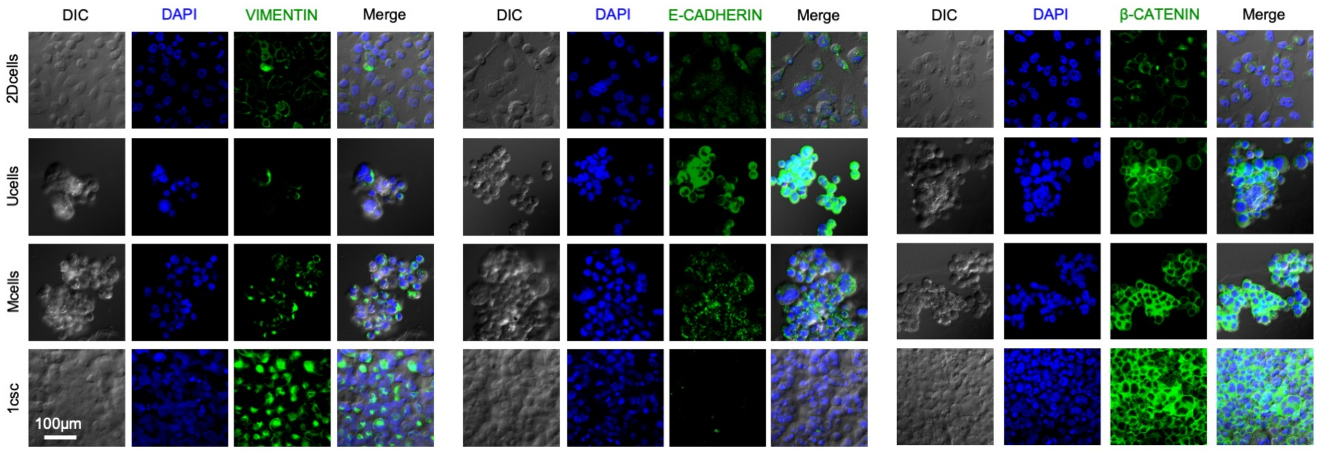

Figure S29. Confocal images showing the expression of epithelial-mesenchymal transition (EMT) markers. The expression of VIMENTIN and β-CATENIN is upregulated and the expression of E-CADHERIN is downregulated in the 1csc colony cells, compared with cells of the other three groups. Increased VIMENTIN and β-CATENIN expression positively correlates with augmented invasiveness and metastasis. Suppression of E-CADHERIN may lead to mesenchymal phenotype, increased cell migration and invasion, as well as metastasis. The data suggest that the 1csc colony cells have higher ability to form metastatic tumors than cells from the 2Dcells, Ucells, and Mcells culture methods.

a

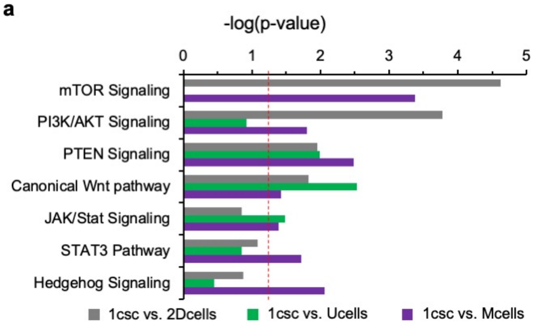

b

Canonical Pathway	Ocscs v.s. 2Dcells	Ocscs v.s. Ucells	Ocscs v.s. Mcells
Oxidative Phosphorylation	17.17	23.15	6.66
Mitochondrial Dysfunction	15.94	20.13	8.36
eIF2 Signaling	15.45	11.10	0.00
Protein Ubiquitination Pathway	5.28	6.03	6.57
Molecular Mechanisms of Cancer	2.72	5.32	4.93
Regulation of eIF4 and p70S6K Signaling	6.76	4.92	0.88
Nucleotide Excision Repair Pathway	3.63	5.10	3.01
Cell Cycle: G1/S Checkpoint Regulation	3.74	3.22	4.13
Cyclins and Cell Cycle Regulation	3.90	3.10	3.31
D-myo-inositol (1,4,5)-Trisphosphate Biosynthesis	2.80	3.57	3.43
Chronic Myeloid Leukemia Signaling	2.35	4.61	2.80
Pancreatic Adenocarcinoma Signaling	0.80	3.72	4.64
Mitotic Roles of Polo-Like Kinase	5.13	2.84	1.17
Aldosterone Signaling in Epithelial Cells	1.62	2.54	4.54
HGF Signaling	0.98	3.25	4.19
CD27 Signaling in Lymphocytes	2.84	3.08	2.21
mTOR Signaling	4.62	3.38	0.00
NRF2-mediated Oxidative Stress Response	1.02	2.65	4.00
Aryl Hydrocarbon Receptor Signaling	2.05	2.14	3.42
Unfolded protein response	1.29	2.39	3.89
Huntington's Disease Signaling	1.19	4.12	2.10
Rac Signaling	1.75	3.33	2.25
Estrogen Receptor Signaling	2.61	2.97	1.71
Superpathway of Inositol Phosphate Compounds	1.09	1.66	4.44
Superpathway of Cholesterol Biosynthesis	4.73	1.55	0.83
p53 Signaling	0.70	2.44	3.62
Cell Cycle Regulation by BTG Family Proteins	3.10	1.93	1.72
Axonal Guidance Signaling	2.53	2.62	1.42
Adipogenesis pathway	0.99	2.37	3.20
PI3K/AKT Signaling	3.78	1.80	0.92
PTEN Signaling	1.95	2.49	1.99
Assembly of RNA Polymerase II Complex	1.89	1.75	2.73
TNFR1 Signaling	3.56	1.77	0.57
RANK Signaling in Osteoclasts	0.97	2.23	2.68
Wnt/Ca+ pathway	1.82	1.43	2.53
Telomerase Signaling	0.83	3.00	1.82
Role of CHK Proteins in Cell Cycle Checkpoint Control	2.62	1.81	1.17
1D-myo-inositol Hexakisphosphate Biosynthesis II	1.75	2.41	1.44
2-oxobutanoate Degradation I	2.61	1.56	1.35
Hereditary Breast Cancer Signaling	1.77	1.86	1.88
Ketolysis	1.53	2.49	1.36
PPARα/RXRα Activation	0.76	2.68	1.86
Apoptosis Signaling	3.42	1.46	0.33
PI3K Signaling in B Lymphocytes	2.01	1.30	1.81
D-myo-inositol-5-phosphate Metabolism	0.57	1.11	3.42
Glucocorticoid Receptor Signaling	1.11	1.56	2.44
3-phosphoinositide Biosynthesis	0.53	0.79	3.77
ErbB2-ErbB3 Signaling	1.47	0.97	2.54
Small Cell Lung Cancer Signaling	1.56	1.77	1.63
Superpathway of D-myo-inositol (1,4,5)-trisphosphate Metabolism	1.00	2.35	1.40

Figure S30. Ingenuity pathway analysis (IPA) of the differentially regulated signaling pathways between the 1csc colony cells and cells from 2Dcells, Ucells, and Mcells groups. **a**, Top 7 differentially expressed stemness-related pathways. The red dashed line indicates the cutoff of the *p* value ($p < 0.05$) for statistical significance. **b**, A list of the canonical pathways that are differentially regulated in the 1csc colony cells in comparison to cells from 2Dcells, Ucells, and Mcells groups, showing that the energy metabolism is one of the most significantly altered pathways.

Canonical Pathway	1csc *In vitro* vs. *in vivo*
Oxidative Phosphorylation	26.87
Mitochondrial Dysfunction	26.80
Protein Ubiquitination Pathway	21.14
EIF2 Signaling	15.36
Regulation of eIF4 and p70S6K Signaling	7.96
Nucleotide Excision Repair Pathway	7.74
tRNA Charging	7.68
Fatty Acid β-oxidation I	6.72
Superpathway of Cholesterol Biosynthesis	5.93
Mitotic Roles of Polo-Like Kinase	5.74
Assembly of RNA Polymerase II Complex	5.62
phagosome maturation	5.43
Hypoxia Signaling in the Cardiovascular System	4.65
Unfolded protein response	4.52
Cell Cycle Control of Chromosomal Replication	4.36
Endoplasmic Reticulum Stress Pathway	4.35
NRF2-mediated Oxidative Stress Response	4.33
Superpathway of Methionine Degradation	4.18
Superpathway of Geranylgeranyldiphosphate Biosynthesis I (via Mevalonate)	4.16
Mismatch Repair in Eukaryotes	4.16
Purine Nucleotides De Novo Biosynthesis II	4.10
Cell Cycle: G2/M DNA Damage Checkpoint Regulation	4.04
TCA Cycle II (Eukaryotic)	3.84
2-oxobutanoate Degradation I	3.81
Ketolysis	3.80
mTOR Signaling	3.71
Mevalonate Pathway I	3.69
Glutaryl-CoA Degradation	3.69
Ketogenesis	3.47
Stearate Biosynthesis I (Animals)	3.44
Role of CHK Proteins in Cell Cycle Checkpoint Control	3.25
Ethanol Degradation II	2.28
Serotonin Degradation	0.27
Pyrimidine Deoxyribonucleotides De Novo Biosynthesis I	3.14
Actin Nucleation by ARP-WASP Complex	3.13
Estrogen Receptor Signaling	3.11
UDP-N-acetyl-D-glucosamine Biosynthesis II	3.10
Isoleucine Degradation I	3.05
Methylmalonyl Pathway	3.05
Hereditary Breast Cancer Signaling	2.97
Telomere Extension by Telomerase	2.79
Polyamine Regulation in Colon Cancer	2.69
Purine Nucleotides Degradation II (Aerobic)	2.62
RAN Signaling	2.56
Guanosine Nucleotides Degradation III	2.52
Retinol Biosynthesis	0.56
Tryptophan Degradation III (Eukaryotic)	2.43
Rapoport-Luebering Glycolytic Shunt	2.41
ATM Signaling	2.41
Tumoricidal Function of Hepatic Natural Killer Cells	2.36
Urate Biosynthesis/Inosine 5'-phosphate Degradation	2.29

Figure S31. A list of the canonical pathways, obtained from the IPA analysis, that are differentially expressed in the *in vitro* 1csc colony cells before injection into mice versus cells in the G1 tumors grown form the *in vitro* 1csc colony cells by injecting them into the fat pads of mice for 55 days.

Gene Sets enriched in 1csc (versus 2Dcells, Ucells, Mcells)

NAME	NES	FDR q-val
Oxidative Phosphorylation	9.666	0
MYC Targets V1	9.508	0
E2F Targets	6.45	0
DNA Repair	5.423	0
Fatty Acid Metabolism	5.246	0
G2M Checkpoint	4.897	0
MTORC1 Signaling	4.367	0
Adipogenesis	4.22	0
Protein Secretion	3.311	0
MYC Targets V2	3.199	0
Peroxisome	2.992	0
Glycolysis	2.837	0
Xenobiotic Metabolism	2.756	0
Interferon Alpha Response	2.745	0
Interferon Gamma Response	2.71	0
Reactive Oxygen Species Pathway	2.482	0
Unfolded Protein Response	2.192	0.003
KRAS Signaling Up	2.163	0.003
Complement	2.149	0.003
UV Response Up	2.039	0.005
Bile Acid Metabolism	1.896	0.012
Apoptosis	1.764	0.026
PI3K AKT MTOR Signaling	1.626	0.049

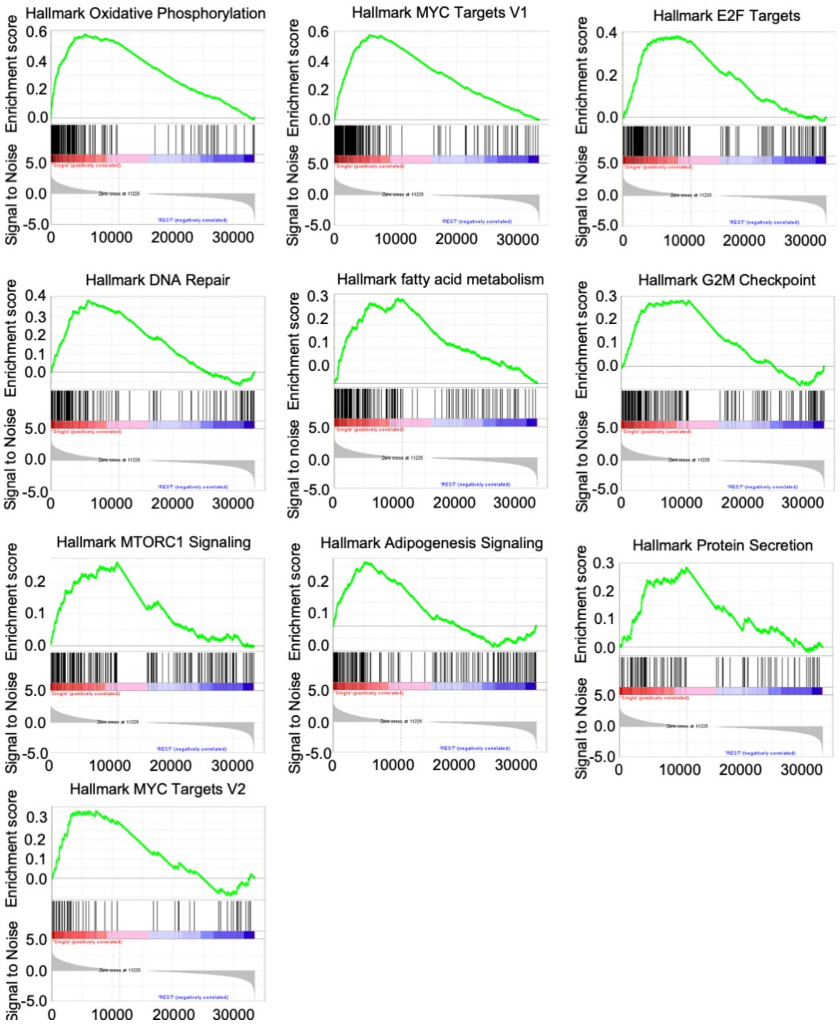

Figure S32. A list of the 23 gene sets enriched in the 1csc colony cells obtained by gene set enrichment analysis (GSEA). Enrichment score (ES) plots of eight of the top 10 gene sets are given and hallmarks of the remaining two (fatty acid metabolism and adipogenesis) are shown in **Figure 6c**.

Gene Sets enriched in 1csc *in vitro* vs. *in vivo*		
NAME	NES	FDR q-val
Oxidative Phosphorylation	11.649	0
Myc Targets v1	10.920	0
E2f Targets	9.330	0
Mtorc1 Signaling	9.285	0
G2M Checkpoint	7.896	0
DNA Repair	7.135	0
Protein Secretion	6.995	0
Unfolded Protein Response	6.723	0
Adipogenesis	6.598	0
Glycolysis	6.365	0
TNFa Signaling via NFKb	6.172	0
Fatty Acid Metabolism	6.167	0
P53 Pathway	6.068	0
Mitotic Spindle	5.163	0
Heme Metabolism	5.129	0
Apoptosis	5.113	0
Hypoxia	4.797	0
Uv Response Up	4.648	0
Cholesterol Homeostasis	4.477	0
Androgen Response	4.395	0
Peroxisome	4.360	0
Xenobiotic Metabolism	4.136	0
PI3k Akt Mtor Signaling	4.116	0
Interferon Gamma Response	4.066	0
Myc Targets v2	3.930	0
Complement	3.925	0
Interferon Alpha Response	3.871	0
Estrogen Response Late	3.726	0
Reactive Oxigen Species Pathway	3.498	0
Il2 Stat5 Signaling	3.250	0
Estrogen Response Early	3.130	0
Uv Response Dn	2.850	0
TGF Beta Signaling	2.824	0
Inflammatory Response	2.672	0
KRAS Signaling Up	2.651	0
Bile Acid Metabolism	2.544	1.35E-04
Il6 JAK Stat3 Signaling	2.405	4.26E-04
Allograft Rejection	1.878	0.011236
Epithelial Mesenchymal Transition	1.828	0.014931

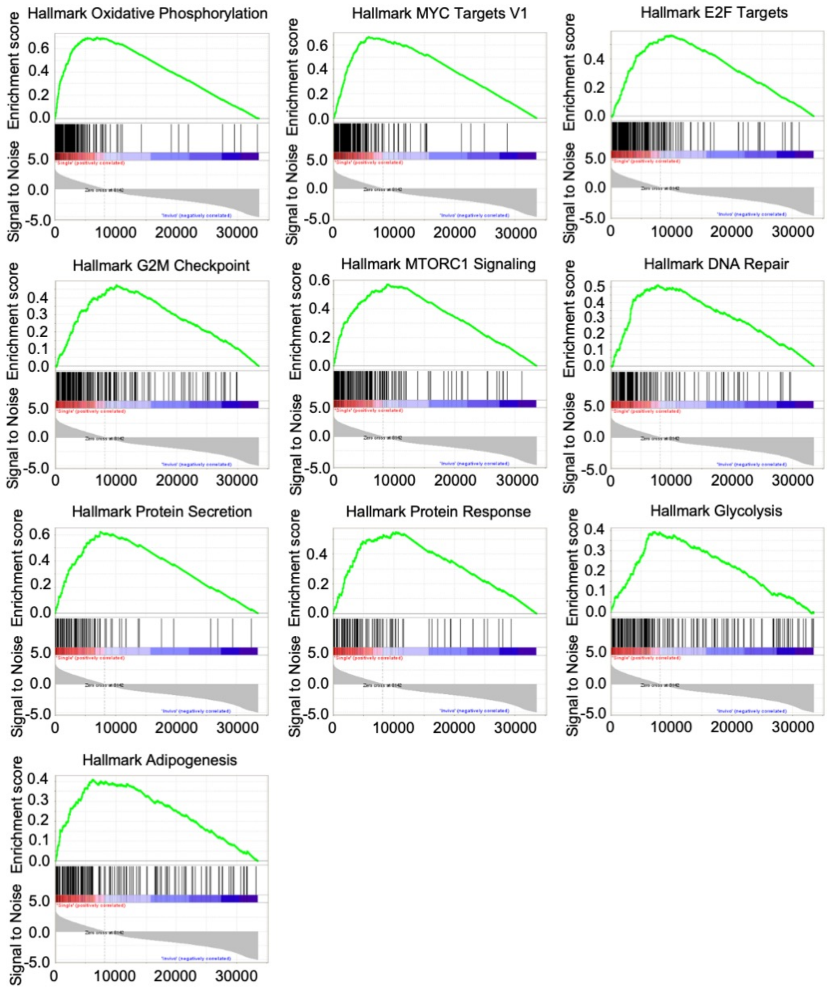

Figure S33. A list of the most enriched 39 gene sets in the 1csc colony cells when compared to the G1 *in vivo* tumors of the 1csc group. Enrichment score (ES) plots of the top 10 gene sets are also given.

Table S1. Detailed information on the amount and quality of the RNAs extracted from cells of the 2Dcells, Ucells, Mcells, and 1csc groups.

Sample ID	Group	Sample Type	Species	Reference Genome	RNA Integrity Number	Concentration (ng µl^{-1})	Volume (µl)	Extraction method	A260/280
1	2Dcells	RNA	Human	Human	9.8	1263	>20	Qiagen RNAeasy plus mini kit	2.06
2	2Dcells	RNA	Human	Human	9.9	1652	>20	Qiagen RNAeasy plus mini kit	2.03
3	2Dcells	RNA	Human	Human	9.9	820.7	>20	Qiagen RNAeasy plus mini kit	2.03
4	Ucells	RNA	Human	Human	10	13.5	>20	Qiagen RNAeasy plus mini kit	1.99
5	Ucells	RNA	Human	Human	10	1403	>20	Qiagen RNAeasy plus mini kit	2.01
6	Ucells	RNA	Human	Human	9.9	33.9	>20	Qiagen RNAeasy plus mini kit	2.03
7	Mcells	RNA	Human	Human	9.5	64.4	>20	Qiagen RNAeasy plus mini kit	1.98
8	Mcells	RNA	Human	Human	10	123	>20	Qiagen RNAeasy plus mini kit	1.94
9	Mcells	RNA	Human	Human	10	92.6	>20	Qiagen RNAeasy plus mini kit	1.9
10	1csc	RNA	Human	Human	9.3	26.7	~11	Ambion Pico Pure RNA isolation kit	1.76
11	1csc	RNA	Human	Human	9.8	37.1	~11	Ambion Pico Pure RNA isolation kit	1.78
12	1csc	RNA	Human	Human	9.5	93.7	~11	Ambion Pico Pure RNA isolation kit	1.82
